# A new microtubule-stabilizing agent shows potent antiviral effects against African swine fever virus with no cytotoxicity

**DOI:** 10.1080/22221751.2021.1902751

**Published:** 2021-03-12

**Authors:** Samvel Sirakanyan, Erik Arabyan, Astghik Hakobyan, Tamara Hakobyan, Garri Chilingaryan, Harutyun Sahakyan, Arsen Sargsyan, Grigor Arakelov, Karen Nazaryan, Roza Izmailyan, Liana Abroyan, Zaven Karalyan, Elina Arakelova, Elmira Hakobyan, Anush Hovakimyan, Andre Serobian, Marco Neves, João Ferreira, Fernando Ferreira, Hovakim Zakaryan

**Affiliations:** a Scientific Technological Center of Organic and Pharmaceutical Chemistry of NAS, Institute of Fine Organic Chemistry of A.L. Mnjoyan, Yerevan, Armenia; b Group of Antiviral Defense Mechanisms, Institute of Molecular Biology of NAS, Yerevan, Armenia; c Russian-Armenian University, Yerevan, Armenia; d Department of Medical Biology, Yerevan State Medical University, Yerevan, Armenia; e Advanced Solutions Center, Foundation for Armenian Science and Technology, Yerevan, Armenia; f Instituto de Medicina Molecular, Faculdade de Medicina, Universidade de Lisboa, Lisboa, Portugal; g Centro de Investigação Interdisciplinar em Sanidade Animal, Faculdade de Medicina Veterinária, Universidade de Lisboa, Avenida da Universidade Técnica, Lisboa, Portugal; h Denovo Sciences, Yerevan, Armenia

**Keywords:** African swine fever virus, microtubule, tubulin, antivirals, screening

## Abstract

African swine fever virus (ASFV) is the causal agent of a fatal disease of domestic swine for which no effective antiviral drugs are available. Recently, it has been shown that microtubule-targeting agents hamper the infection cycle of different viruses. In this study, we conducted in silico screening against the colchicine binding site (CBS) of tubulin and found three new compounds with anti-ASFV activity. The most promising antiviral compound (6b) reduced ASFV replication in a dose-dependent manner (IC50 = 19.5 μM) with no cellular (CC50 > 500 μM) and animal toxicity (up to 100 mg/kg). Results also revealed that compound 6b interfered with ASFV attachment, internalization and egress, with time-of-addition assays, showing that compound 6b has higher antiviral effects when added within 2–8 h post-infection. This compound significantly inhibited viral DNA replication and disrupted viral protein synthesis. Experiments with ASFV-infected porcine macrophages disclosed that antiviral effects of the compound 6b were similar to its effects in Vero cells. Tubulin polymerization assay and confocal microscopy demonstrated that compound 6b promoted tubulin polymerization, acting as a microtubule-stabilizing, rather than a destabilizing agent in cells. In conclusion, this work emphasizes the idea that microtubules can be targets for drug development against ASFV.

## Introduction

African swine fever virus (ASFV) is the causative agent of a devastating and economically significant disease of domestic pig. There are no effective vaccines or antiviral agents against ASFV, and therefore current control methods rely on quarantine and culling of animals in affected areas [1,2]. Although current control methods are in place, we have seen the spread of the ASFV across Europe along with recent outbreaks in China where millions of pigs have been culled in an effort to halt its spread in 32 provinces since August 2018. These examples of continuing spread of the ASFV, considering current control measures, highlights the need for development of improved control strategies that include vaccines and antiviral drugs.

Based on their inhibitory mechanisms, antiviral agents can be divided into two groups: (i) inhibitors that target the viral proteins (direct-acting antivirals) and (ii) inhibitors that impair host cell factors that are essential for the viral life cycle (host-targeting antivirals). Although most of the current antiviral drugs affect the viruses themselves, an ever-growing number of antiviral agents targeting the host cell proteins are reported. For instance, Maraviroc (approved for the treatment of HIV-1 infection) acts as an antagonist of the CCR5 receptor [3]. As another example, a non-immunosuppressive inhibitor of cyclophilin A, Alisporivir, is currently being tested (phase III trials) for antiviral activity against hepatitis C virus [4]. Thus, the targeting of host factors can significantly increase the pool of druggable targets and speed up the drug discovery process.

Previous studies showed that ASFV replication depends on the host cell microtubules. Incoming ASFV particles hijack the microtubule motor complex to reach the perinuclear region (viral factory) where the viral replication occurs [5]. Next, recruitment of *de novo* synthesized ASFV proteins to the viral factories are also dependent on microtubules [6]. Finally, the mechanism of ASFV egress involves the transport of new particles from the virus factories to the cell surface through a microtubule-mediated mechanism [7,8]. In recent work, we reported the inhibitory effect of plant-derived genkwanin on ASFV infection [9]. *In silico* simulations showed that genkwanin can bind to the intermediate domain of beta-tubulin known as the colchicine binding site (CBS). Since microtubules are assembled from dimers of alpha- and beta-tubulin, we hypothesized that microtubule-targeting molecules may successfully disrupt ASFV infection through interfering with the dynamics of microtubules. This hypothesis prompted us to explore microtubules as antiviral targets for ASFV.

In this study, we first showed the inhibitory effect of well-known microtubule-targeting agents on ASFV infection *in vitro*. Then, using the crystallographic structure of tubulin, we conducted an *in silico* screening of small molecules to identify new compounds that can interact with tubulin and inhibit ASFV infection. From this screening, we found a compound that demonstrated microtubule-stabilizing effects and reduced ASFV infection *in vitro* with no significant host cytotoxicity. Our findings support the hypothesis that microtubules can be considered as attractive host targets in antiviral drug discovery against ASFV.

## Materials and methods

### Cells, viruses and drugs

Vero (African green monkey kidney) cells were maintained at 37°C in Eagle’s minimum essential medium (EMEM) (Lonza, Switzerland) supplemented with 10% of fetal bovine serum (Lonza, Switzerland), 2 mM L-glutamine (Lonza, Switzerland), 100 IU/ml penicillin (Reyoung, China) and 100 μg/ml streptomycin (Arterium, Ukraine). In experiments with Vero cells, the Vero-adapted ASFV Ba71 V strain was used and the viral titration was measured by cytopathic effect (CPE-based) assay on Vero cells. The titre was calculated by Spearman-Kärber endpoint method and expressed as TCID_50_/ml.

Preparation of porcine alveolar macrophages was done as previously described [10]. Alveolar cells were cultured at 37°C in Dulbecco’s Modified Eagle’s Medium (Sigma-Aldrich, Germany) supplemented with 10% of fetal bovine serum, 2 mM L-glutamine, 100 IU/ml penicillin and 100 μg/ml streptomycin. Porcine alveolar macrophages were infected by the virulent ASFV Armenia/07 strain. The titration of this strain was performed by hemadsorption (HAD) assay as previously described [10]. The titre was expressed as HADU_50_/ml.

Colchicine, nocodazole, vinblastine and paclitaxel were purchased from Sigma-Aldrich (Germany). They were dissolved in dimethyl sulfoxide (DMSO) as 20 mM stock and resuspended in EMEM. At the time of experiments, dilutions in cell culture medium were performed with the final concentration of DMSO not exceeding 1% (v/v).

### Computational experiments

The virtual screen was perfomed with a four-dimensional (4D) docking method implemented in ICM-pro program [11]. Crystallographic structures of tubulin with different small molecules in the colchicine binding site were obtained from the Pocketome database (Pocketome ID: TBB5_P1 PDB ID: 3ryh, 4iij, 4o4h, 4x1i, 4x1k, 4yj2, 5c8y, 5ca1, 5cb4, 5eyp, 5h74, 5jcb, 5jh7, 5njh, 5o7a, 5ylj, 6fkj, 6gvm, 3ryc, 3ut5, 4drx, 4hna, 4i4t, 4i50, 4i55, 4o2b, 4o4i, 4tuy, 4tv8, 4tv9, 4wbn, 4x1y, 4x20, 4zhq, 5bmv, 5eib, 5ezy) [12]. The defined site included 42 amino acids and 37 tubulin structures were used for generating 4D docking maps. The ligands library (Scientific and Technological Center of Organic and Pharmaceutical Chemistry, National Academy of Sciences, Armenia) was assembled in SDF format and the MMFF94 force field was used for the optimization of ligands and assignment of atomic charges [13]. The virtual screening was conducted with fully flexible ligand sampling including the sampling of linear chiral centres and cis/trans conformations.

The molecular dynamics simulations were carried out using AMBER18 with ff14SB force field for protein and GAFF for the ligand parameterization according to the AM1-BCC scheme order to calculate the atomic point charges [14–16]. The docked conformation of tubulin with compound **6b** was used as a starting position. The complex was solvated with TIP3P water and Na^+^/Cl^−^ ions at 150 mM concentration [17]. Afterward, the complex was minimized and equilibrated gradually releasing position restraints during 50 ns. The Monte Carlo barostat with reference pressure at 1 bar and Langevin thermostat with collision frequency (gamma_ln) 2 ps^−1^ to keep the temperature at 310.15 K were used [18,19]. Particle Mesh Ewald (PME) with electrostatic interactions cut off at 1.0 nm was used for the long-range electrostatic interactions. Bonds involving hydrogen were constrained using the SHAKE algorithm and 2fs integration step was used [20]. For evaluation of binding free energy MM/PB(GB)SA calculations were performed with MMPBSA.py program [21]. The calculations were conducted with 1000 snapshots collected from all over the trajectory with equal intervals.

### Chemistry

All compounds were synthesized in-house. All details are presented in supplementary materials. Some starting compounds were already reported [22–26].

### Antiviral assays

For primary antiviral screening, Vero cells grown in 24-well cell culture plates (seeding density: 2 × 10^5^ cell/well) were infected with the ASFV Ba71 V strain at a MOI of 0.2 TCID_50_/cell and treated with microtubule-targeting drugs and selected compounds (4 wells per compound) at the indicated concentrations. The infection was allowed to proceed for 24 and 72 h with microtubule-targeting drugs and selected compounds respectively, after which the supernatant was collected and titrated.

For the dose-dependent assay, Vero cells grown in 24-well cell culture plates (seeding density: 2×10^5^ cell/well) and macrophages (seeding concentration: 4 × 10^4^ cell/well) in 96-well plates were incubated with ASFV Ba71 V (0.2 TCID_50_/cell) or ASFV Armenia/07 strain (0.5 HADU_50_/cell). Compound was added at decreasing concentrations (from 100 to 12.5 μM). The virus was collected at 72 h post-infection and titrated.

For the time-of-addition assay, Vero cells grown in 24-well cell culture plates (seeding density: 2 × 10^5^ cell/well) and macrophages (seeding concentration: 4 × 10^4^ cell/well) in 96-well plates were incubated with ASFV Ba71 V (0.2 TCID50/cell) or ASFV Armenia/07 strain (0.5 HADU50/cell). Then, cells were exposed to the compound at 2, 4, 8, 12 and 16 h post-infection, after which the virus was collected at 72 h post-infection and titrated.

The time-of-removal assay was conducted using Vero cells grown in 24-well cell culture plates and infected with ASFV Ba71 V at concentrations indicated above. The compound was added at 1 h post-infection and discarded at incubation time intervals of 2, 4, 6, 8 h post-infection by washing cells with 1× PBS. The virus was collected and titrated at 72 h post-infection.

For the attachment assay, Vero cells grown in a 24-well cell culture plates (seeding density: 2 × 10^5^ cell/well) were incubated with ASFV (0.2 TCID_50_/cell) and compound at 4°C for 1 h to allow virus binding but prevent viral internalization. The unbound virus and compound were then discarded by thoroughly washing the cells with 1× PBS and then adding EMEM containing 3% FBS. The plates were then into a 37°C incubator for 72 h.

For the internalization assay, cells were incubated with ASFV at 4°C for 1 h. Then, the unbound virus was discarded by thoroughly washing the cells with 1× PBS and incubating them at 37°C to allow virus entry to proceed. The tested compound was added at 0 h and removed at 1 h during the incubation at 37°C. Then, the cells were thoroughly washed with 1× PBS before fresh EMEM was added. The time point at which cells were shifted to 37°C was considered as 0 h. The virus was collected and titrated after 72 h.

For virus egress assay, Vero cells grown in 24-well cell culture plates (seeding density: 2 × 10^5^ cell/well) plates were incubated with ASFV Ba71 V (0.2 TCID_50_/cell). At 16 h post-infection, ASFV-infected cells were treated with the compound for 1 h. Then, control and treated cells were washed three times with 1× PBS and fresh medium was added. At 24 h post-infection, when the first cycle of ASFV was completed and new viral particles were released, the supernatant was harvested and titrated. The same supernatant was used to quantify the amount of viral major capsid protein by ELISA kit (Ingenasa, Spain). The experiment was conducted according to the manufacturer’s protocol. The optical density (OD) was measured at 405 nm by the BioTek Epoch2 (USA) microplate reader.

For the comet tail (spreading) assay, sub-confluent Vero cell monolayers in 6-well culture plates were pretreated with DMSO or compound at concentrations of 50 and 100 μM for 20 min at 37°C and subsequently infected with ASFV Ba71 V (7 × 10 TCID_50_/well). After 1 h post-infection, the inoculum was removed and cells were cultured in liquid growth medium (to allow virus spread) containing DMSO or tested compound throughout the experiment. At 48 h post-infection, cells were fixed, stained with crystal violet, and photographed.

For the virucidal assay, the virus suspension containing 2 × 10^5^ TCID_50_ particles was incubated with a volume solution of compound (100 μM), for 1 h at 37°C. Then, Vero cells in 96-well cell culture plates (seeding density: 2 × 10^4^ cell/well) were infected with the 20-fold diluted treated viral suspension to eliminate the potential effects of compound on ASFV infection. The virus was collected and titrated after 72 h.

### Cytotoxicity

The cytotoxicity of the tested compounds was evaluated in Vero cells and porcine alveolar macrophages by MTT assay. Confluent cells in 96-well cell culture plates (seeding density: 2 × 10^4^ cell/well) were treated with increasing concentrations of compound ranging from 6.25 to 100 μM. Treated cells were incubated for 72 h at 37°C in 5% CO_2_. After incubation, medium was removed, cells were washed with cold PBS and MTT solution (Sigma-Aldrich, Germany) was added. Cells were incubated at 37°C for 2 h after adding MTT solution followed by purple formazan extraction by MTT solvent (DMSO). The colorimetric measurements were performed on a microplate reader at 570 nm (BioTek Epoch2, USA). The percentage of viable cells were calculated for each concentration as [(OD_T_/OD_C_) × 100], where OD_T_ and OD_C_ correspond to the absorbance of treated and control cells respectively. The 50% cell cytotoxicity (CC_50_) was determined as the concentration of the compound, which causes 50% cellular death. The cytotoxicity of the final concentration of DMSO was also measured.

### Quantification of DNA in ASFV factories

Vero cells were grown on coverslips (3  ×  10^5^ cell/well) in 12-well plates and infected with ASFV Ba71 V (2 TCID_50_/cell), and exposed to the compound (100 μM) at 8 h post-infection. At 16 hpi, cells were fixed in a 96% ethanol solution for 30 min and stained in fresh Schiff’s reagent (DNA hydrolysis in 5N hydrochloric acid, 60 min at 22°C) by the Feulgen method. The cytometric equivalent of DNA content of viral factories was measured by computer-equipped microscope-cytometer SMP 05 (Carl Zeiss, Germany) at 575 nm and expressed as an Integrated Optical Density (IOD). The measurement was carried out for 100 viral factories per sample.

### Confocal microscopy

All details are presented in supplementary materials.

### Western blotting analysis

Vero cells grown in 30 mm dishes were infected with the ASFV Ba71V at different MOI (2 or 5), after the adsorption period (1 h). Following this step and before protein extraction, mock-infected and ASFV-infected cells either exposed or not to the compound were washed twice with PBS and processed as previously reported [27]. Briefly, the clarified whole-cell lysates harvested at 16 hpi were subjected to SDS-PAGE gel electrophoresis and transferred by electroblotting. The membranes were incubated with specific primary antibodies (at room temperature, 1 h), followed by a wash step with PBST (3 ×  10 min), and thereafter incubated with appropriate secondary antibodies conjugated with HRP, for 1 h at RT. Finally, protein detection was performed by using a chemiluminescence detection kit (Pierce^®^ ECL Western Blotting Substrate, Thermo Scientific), on Amersham Hyperfilm ECL (GE Healthcare, Piscataway, NJ, USA). For the blot analysis, three primary antibodies (in-house mouse anti-ASFV-pA104R, 1:100; in-house mouse anti-ASFV-pI215L, 1:100; anti-β-actin, 1:1000, 13E5, Cell Signaling Technology) and two HRP-conjugated secondary antibodies were used (anti-mouse IgG, 1:30000, 1010–05; anti-rabbit IgG, 1:10000, 4010–05; both from Southern Biotech). All antibody dilutions were performed in blocking solution and incubated according to the manufacturer’s recommendations.

### Flow cytometry

For the flow cytometry analysis, cells were stained for DNA (with DAPI), ASFV and anti-ASFV mouse antibody pA104R. Cytometry acquisition was performed on a BD LSRFortessa 2 cell analyser (BD Biosciences, San Jose, CA, USA). Data analysis, such as mean fluorescence intensity (MFI) measurements, was performed using FlowJo X10.6.0 software.

### Histology and blood cell analysis

All details are presented in supplementary materials.

### Statistics

The CC_50_ was calculated by a linear regression analysis of dose–response curves generated from the data. The IC_50_ was calculated by a nonlinear regression analysis of dose–response curves generated from the data. Data are expressed as mean ± SD of three independent experiments. Data was analysed by Student’s t-test. A *P* < 0.05 was considered to be statistically significant.

## Results

### Anti-ASFV activity of microtubule-targeting agents

**Figure 1.**
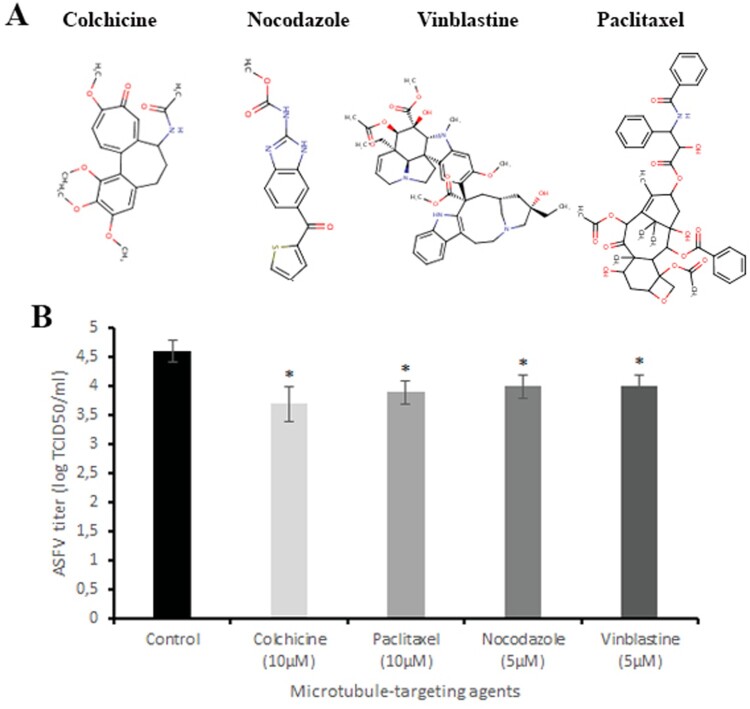
Antiviral effect of microtubule-targeting agents on ASFV infection *in vitro*. (A) The chemical structures of colchicine, nocodazole, vinblastine and paclitaxel. (B) ASFV titre in Vero cells upon treatment with microtubule-targeting agents at indicated concentrations. This data represents the mean (±SD) of three independent experiments (*n* = 3). Significant differences compared to control are denoted by *(*P* < 0.05).

To examine whether microtubule-targeting agents may disrupt ASFV infection, Vero cells were infected with the ASFV and exposed to colchicine (10 µM), paclitaxel (10 µM), nocodazole (5 µM) and vinblastine (5 µM) (Figure 1A) at non-toxic concentrations for 24 h (Figure 1S). After 24 h post-infection, the supernatant was collected and titrated. Data from this assay revealed that all tested drugs reduced viral titre from 4.6 ± 0.19 log TCID_50_/ml (control) to 3.7 ± 0.3 log TCID_50_/ml (colchicine, *P* < 0.05), 3.9 ± 0.2 log TCID_50_/ml (paclitaxel, *P* < 0.05), 4 ± 0.2 log TCID_50_/ml (nocodazole and vinblastine, *P* < 0.05) (Figure 1B), supporting the idea that microtubules could represent interesting antiviral host targets against the ASFV. However, since prolonged exposure (>24 h) of cells to these drugs at indicated concentrations led to increased cytotoxicity, their use as anti-ASFV agents is considered undesirable. Therefore, virtual screening analysis was carried out to find new microtubule-targeting agents that maintain their biological activity but have a significant reduction of cytotoxicity.

### Identification of hits through in silico screening

**Figure 2.**
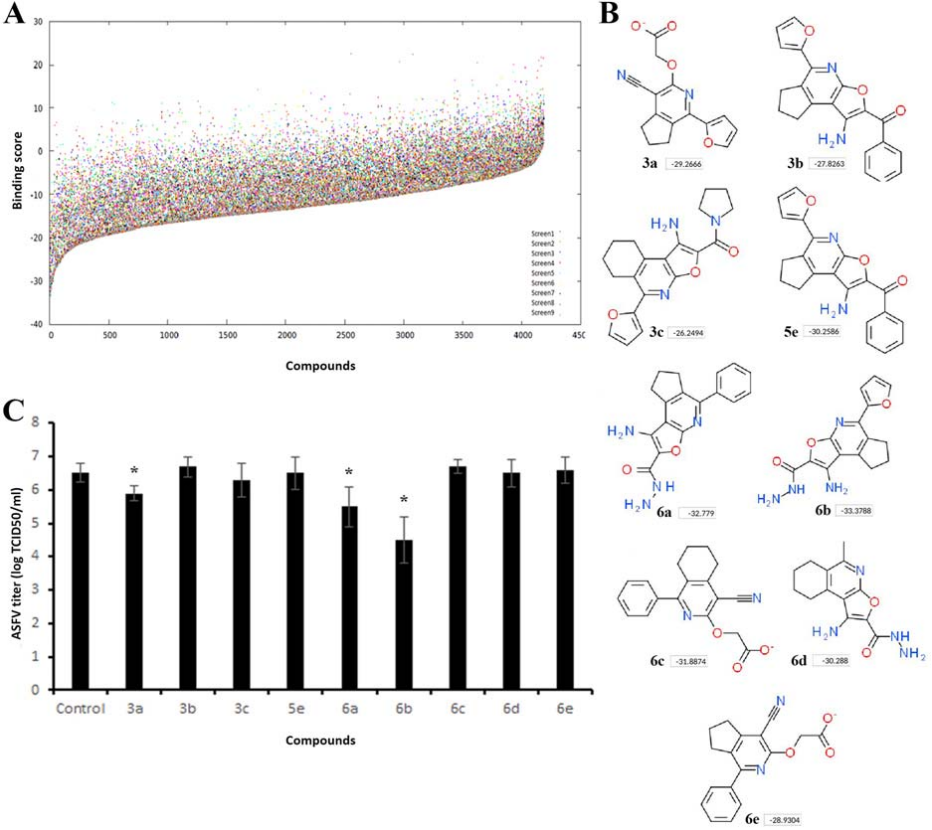
Screening of compounds interacting with the CBS of tubulin. (A) History of docking scores generated during virtual screening run to identify initial hits. Dots indicate individual scores for each compound generated from nine different runs. (B) Nine top scoring compounds selected for antiviral assay. Docking scores are presented in boxes. (C) ASFV titre in Vero cells upon treatment with selected compounds at 100 µM concentration. This data represents the mean (±SD) of three independent experiments (*n* = 3). Significant differences compared to control are denoted by *(*P* < 0.05).

At the molecular level, microtubule-targeting agents have different binding sites on tubulin [28]. For structure-based virtual ligand screening, we used the CBS due to the fact that colchicine reduced the virus yield more than other drugs (Figure 1B). Computational screening based on the 4D docking method was performed on nearly 4200 small molecules (Figure 2A). In general, all compounds were docked between β-sheets S8, S9, and α-helices H7, H8 of the β-tubulin similar to many other CBS inhibitors [29]. The best binding scores were recorded among compounds 6a-d with a aminofuro(thieno)[2,3-b]pyridine-2-carbohydrazide scaffold. We selected 9 compounds with the highest binding scores (Figure 2B) for *in vitro* screening.

### In vitro screening of selected hits

The general procedure for synthesis of compounds and their physicochemical properties are described in supplementary materials. In order to assess whether selected hits were able to inhibit ASFV infection *in vitro*, Vero cells were infected with the ASFV Ba71 V strain and treated with compounds at 100 µM concentration. Figure 2C shows that compound **6b** exerted the highest anti-ASFV activity among the hits tested. It reduced the viral yield from 6.5 ± 0.3 log TCID_50_/ml to 4.5 ± 0.7 log TCID_50_/ml (*P* < 0.05). While the ASFV infection was inhibited by 99% in cells treated with compound **6b** (Figure 2B), compounds **3a** and **6a** also reduced virus growth by nearly 75% and 90%, respectively. Based on these results, we selected compound **6b** for further investigations.

### Antiviral properties of compound **6b**

**Figure 3.**
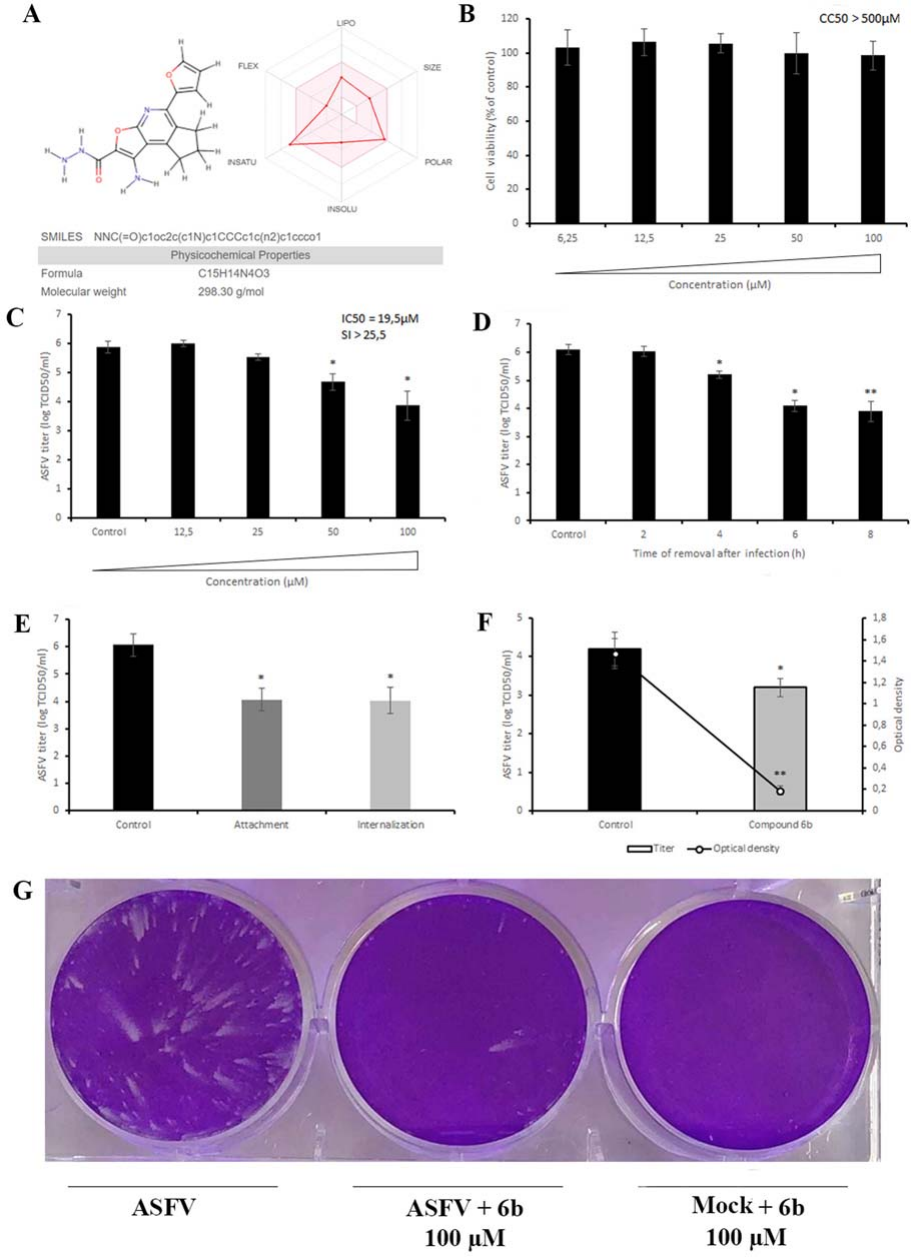
Chemical, cytotoxic and antiviral properties of compound **6b**. (A) Physicochemical properties of tested compound calculated by SwissADME web tool. (B) Cytotoxicity of **6b** on Vero cells evaluated by MTT assay. The CC_50_ was calculated by a linear regression analysis. (C) ASFV yield in Vero cells treated with **6b** at several concentrations. The IC_50_ was calculated by a nonlinear regression. SI = CC_50_/IC_50_. (D) Time-of-removal study of compound **6b** on ASFV infection. The compound was removed at specific intervals of post-infection. (E) Effect of **6b** on ASFV entry into Vero cells. (F) Effect of **6b** on ASFV egress from Vero cells evaluated by titration (bar graph) and ELISA (line graph). (G) ASFV spread in Vero cells treated with compound **6b**. The concentration of tested compound in (D), (E), (F), (G) experiments was 100 µM or 50 µM. This data represents the mean (± SD) of three independent experiments (*n* = 3). Significant differences compared to control are denoted by *(*P* < 0.05) and **(*P* < 0.02).

The molecular weight of compound **6b** (1-Amino-5-(2-furyl)-7,8-dihydro-6*H*-cyclopenta[*d*]furo[2,3-*b*]pyridine-2-carbohydrazide) is 298.3 g/mol. For this compound, lipophilicity, size, polarity, insolubility, insaturation and flexibility are within the suitable physicochemical space for oral bioavailability predicted by using the SwissADME web tool (Figure 3A) [30]. This compound also complies with the Lipinski, Ghose, Veber, Egan, and Muegge rules. The half-life of compound **6b** in serum predicted by ICM software is 1.42 h (Table S1).

We first carried out an MTT (3-(4,5-dimethylthiazol-2-yl)-2,5-diphenyltetrazolium bromide) assay to evaluate the viability of Vero cells in the presence of compound **6b** at different concentrations ranging from 6.25 to 100 µM. As shown in Figure 3B, compound **6b** did not affect cell viability at concentrations up to 100 µM (CC_50_ > 500 µM) after 72 h of incubation, suggesting that its antiviral effect was independent of cellular toxicity. Thus, in all other antiviral assays compound **6b** was used at 100 µM concentration.

We then evaluated the inhibitory activity of compound **6b** at different concentrations in the µM range and showed that it inhibited ASFV replication in a dose-dependent manner with an IC_50_ value of 19.5 µM (Figure 3C). We also conducted time-of-removal studies to determine the optimal treatment duration required for antiviral effects on ASFV replication. For this purpose, the compound was added at 0 h post-infection and removed at specific intervals. When complete cytopathic effect (CPE) was observed in untreated cells (72 h post-infection) the virus was collected and quantified. Gradual declines in viral titres were observed as the interval between infection and compound removal increased (Figure 3D). Treatment with compound **6b** for 8 h resulted in a 2.2 log decrease in viral titre (*P* < 0.02). Since a 2-log decrease was also recorded in primary screening (Figure 2C) and dose-dependent (Figure 3C) assays, we propose that 8 h of treatment is the optimal treatment duration to achieve a strong antiviral effect.

To rule out that compound **6b** has a direct virucidal effect on ASFV, we next pre-incubated the compound with the virus suspension for 1 h and then diluted (20-fold) it to sub-therapeutic concentrations before adding it to Vero cells. Our results showed that **6b** did not influence ASFV infectivity, indicating that this compound is not virucidal (Figure S2).

### Inhibition of ASFV entry

Since microtubules have been shown to play an important role in ASFV entry, we further evaluated the antiviral potential of compound **6b** when it was added at the virus entry stage [5]. To define whether compound **6b** can alter ASFV attachment to Vero cells, the compound was added at 4°C, a condition in which the virus binds to but does not enter cells. Treatment with compound **6b** resulted in a 99% inhibition of virus yield, reducing the virus titre from 6.07 ± 0.5 log TCID_50_/ml to 4.07 ± 0.4 log TCID_50_/ml (*P* < 0.05) (Figure 3E). To determine whether compound **6b** also acts at the internalization stage, it was added immediately after a temperature shift from 4°C to 37°C and removed after 1 h of treatment, to minimize its further effect on other stages of ASFV replication. The titres were reduced from 6.07 ± 0.5 log TCID_50_/ml to 4.03 ± 0.4 log TCID_50_/ml (*P* < 0.05) (Figure 3E). Taken together, these data demonstrated that compound **6b** partially blocked ASFV attachment and internalization steps.

### Inhibition of ASFV egress and spread

It has also been shown that the ASFV transport to the cell periphery is dependent on microtubules [8]. Therefore, to test whether compound **6b** also has an effect on the release of new viral particles, ASFV-infected Vero cells were exposed to compound **6b** at 16 h post-infection, which coincides with the time point when ASFV is transported from the perinuclear assembly site to the cell surface. After 1 h of incubation, the compound was removed and fresh medium was added. At 24 h post-infection, when the first cycle of ASFV was completed, the supernatant was collected to quantify the virus. As shown in Figure 3F, compound **6b** decreased the viral titre by 1 log. Since the compound 6b was added at 16 h post-infection, when it had no chance to affect the assembly process of new ASFV particles [8], we measured the amount of major capsid protein (p72) only in the supernatant in order to define whether this reduction was related to the impaired release of ASFV rather than loss of infectivity. More than an eightfold decrease in the amount of viral p72 was detected (Figure 3F), indicating that the release of viral particles was affected by the treatment with compound **6b**.

As the experiments described above revealed an inhibitory effect on ASFV release, attachment, and internalization, we also hypothesized that compound **6b** should hamper the virus spread. Therefore, we conducted the widely used comet tails assay for determining the effectiveness of our proposed antiviral drug in inhibiting viral spread. Indeed, this assay demonstrated that in the presence of compound **6b** there were a smaller number of plaques and comets compared to untreated cells, thereby unequivocally confirming the inhibitory effect of compound **6b** on the virus spread (Figure 3G).

### Inhibition of viral factories, DNA and protein synthesis

**Figure 4.**
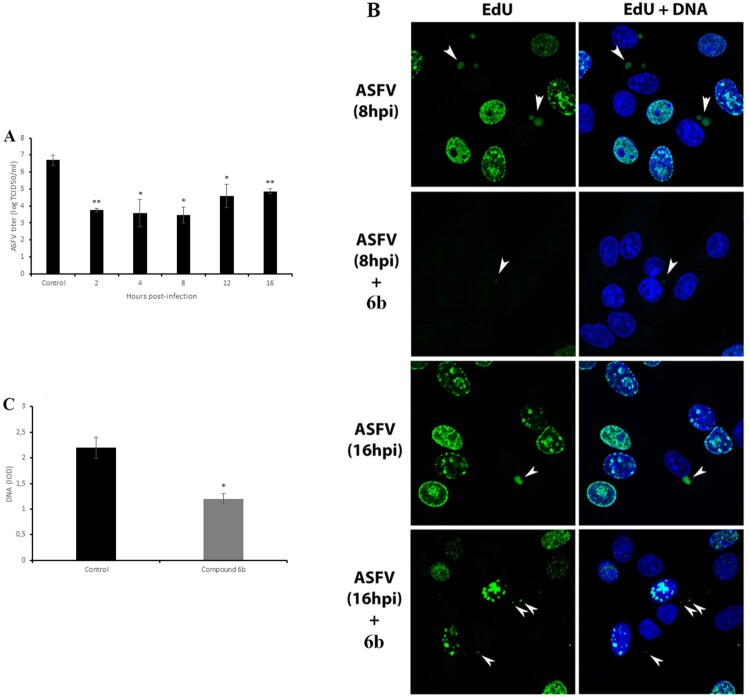
Antiviral effect of tested compound on post-entry stage of ASFV infection and viral factories. (A) Time-of-addition study of compound **6b** on ASFV infection. The compound was added at specific intervals of post-infection. (B) ASFV factories (indicated by arrows) in Vero cells exposed to the thymidine analogue EdU at 8 h and at 16 h post-infection. (C) The DNA content of ASFV factories in cells treated with compound **6b** at 8 h post-infection. In all experiments, the concentration of tested compound was 100 µM. This data represents the mean (±SD) of three independent experiments (*n* = 3). Significant differences compared to control are denoted by *(*P* < 0.05) and **(*P* < 0.02).

The ASFV life cycle may have other potentially vulnerable points where compound **6b** might act. Therefore, the time-of-addition assays were used to determine the specific replication steps of ASFV that can be affected. The compound **6b** was added to ASFV-infected Vero cells at five time points after infection followed by viral quantification at 72 h post-infection. The data indicated that this compound was maximally effective when it was added within 2–8 h post-infection (Figure 4A). It reduced the viral titre from 6.7 ± 0.3 log TCID_50_/ml to 3.5 ± 0.5 log TCID_50_/ml (*P* < 0.05). As 8 h post-infection coincides in time with the formation of ASFV factories, we propose that compound **6b** may affect the formation of viral factories by disrupting the transport of viral proteins and other components along microtubules to the viral factories. As expected, confocal microscopy revealed diminished viral factories in the presence of compound **6b** at 8hpi and at 16hpi (Figure 4B). Furthermore, a significant reduction (43%, *P* < 0.05) in the amount of viral DNA was also observed (Figure 4C) when the tested compound was added at 8 h post-infection.

**Figure 5.**
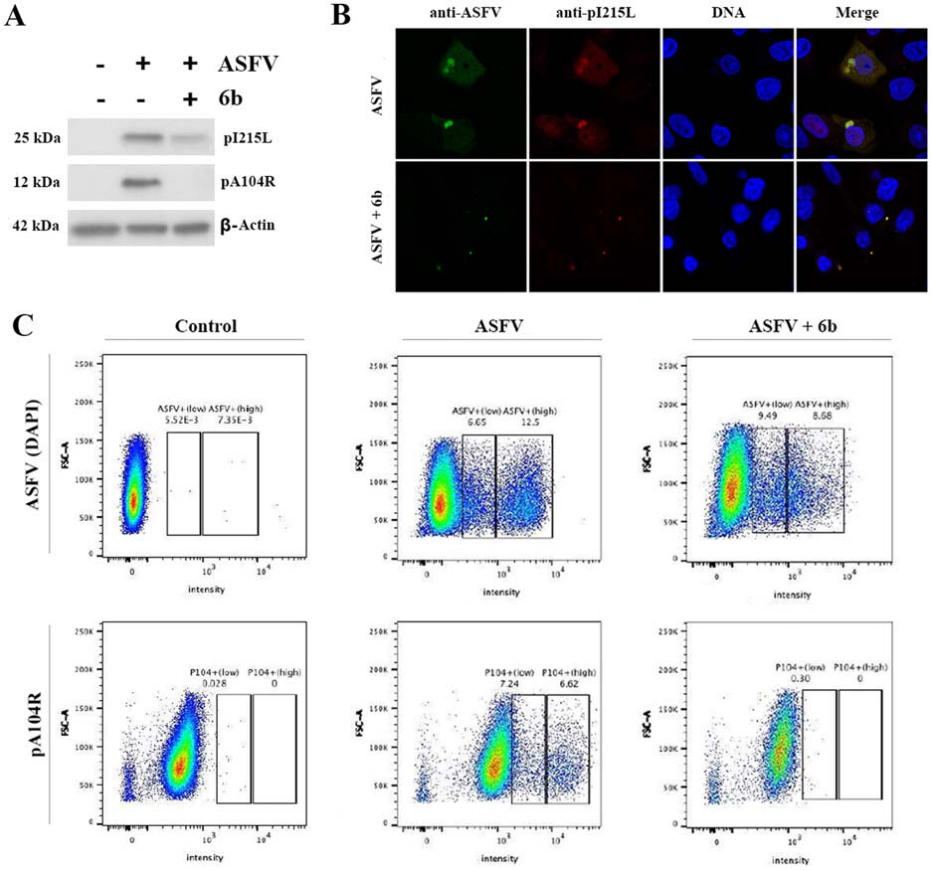
Analysis of ASFV early (pI215L) and late (pA104R) proteins synthesis. (A) Protein synthesis analysed by Western blotting in ASFV-infected Vero cells exposed or not to the 6b compound. β-actin was used as a loading control. (B) Vero cells stained for ASFV proteins (anti-ASFV) and viral pI215L protein at 8 h post-infection. (C) Analysis of pA104R expression and DNA synthesis in mock-infected and ASFV-infected Vero cells exposed or not to the 6b compound, by flow cytometry. In all experiments, compound **6b** (100 µM) was added 1 h post-infection to exclude its inhibitory effect on ASFV entry process.

Knowing that compound **6b** suppressed viral DNA synthesis, we also investigated whether the ASFV protein synthesis was affected by detecting the expression of an early (pI215L) and late (pA104R) viral proteins via western blot, flow cytometry and confocal microscopy techniques at MOI of 5. The results showed that the synthesis of both proteins was significantly reduced following treatment with compound **6b** at MOI of 5 (Figure 5). Similar western blot data were observed at MOI of 2 (data not shown). These data indicate that compound **6b** displays antiviral activity at different stages of ASFV infection.

### Inhibition of ASFV infection in porcine macrophages

**Figure 6.**
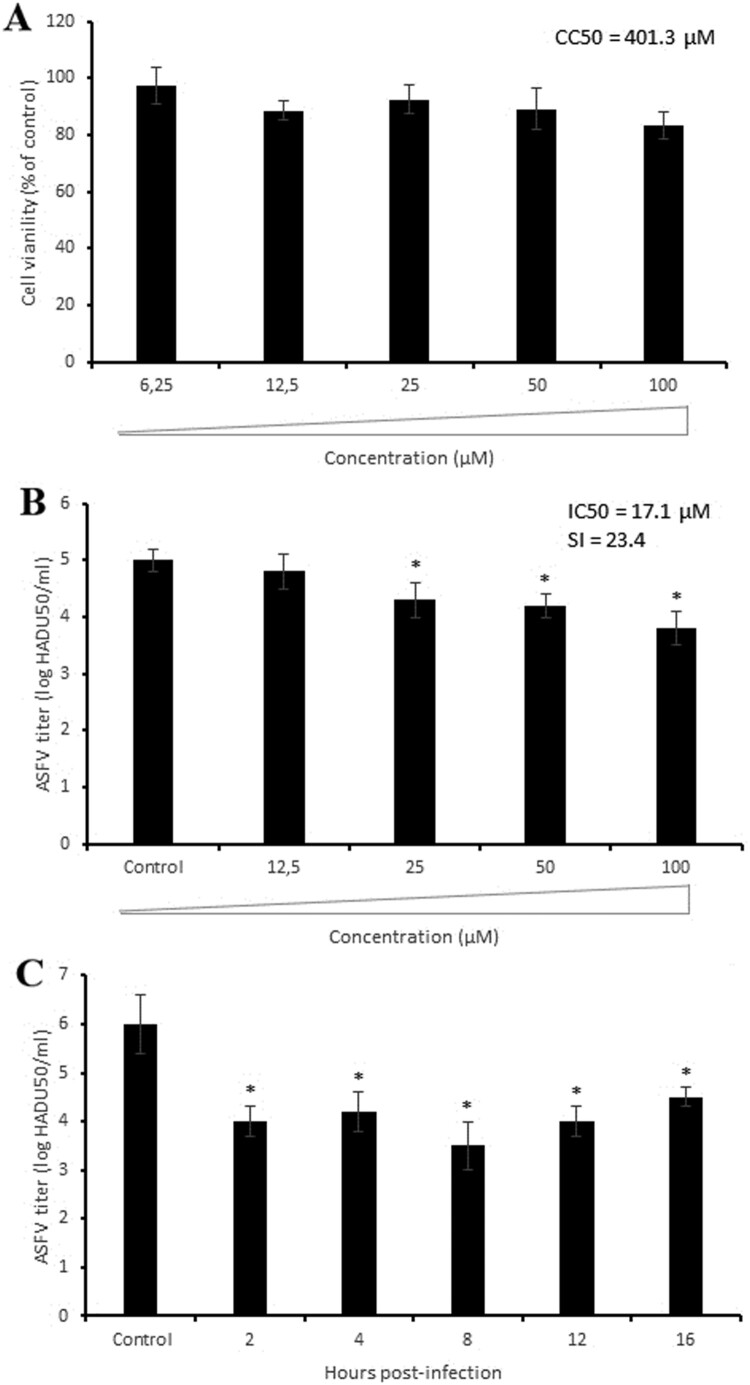
Antiviral activity of compound **6b** against ASFV Armenia/07 strain in porcine macrophages. (A) Cytotoxicity of **6b** on macrophages evaluated by MTT assay. The CC_50_ was calculated by a linear regression analysis. (B) ASFV titre in macrophages treated with **6b** at several concentrations. (C) Time-of-addition study of compound **6b** on ASFV infection in macrophages. The compound (100 µM) was added at specific intervals of post-infection. This data represents the mean (±SD) of three independent experiments (*n* = 3). Significant differences compared to control are denoted by *(*P* < 0.05).

In pigs, monocytes and macrophages are the main host cells for ASFV replication. Therefore, we performed additional antiviral experiments to define the effect of compound **6b** on the virulent ASFV isolate (Armenia/07) in porcine macrophages. Since no macrophage toxicity was seen at concentrations up to 100 µM (Figure 6A), we treated ASFV-infected macrophages with the same concentration of compound **6b** as used in experiments with Vero cells. The antiviral activity was observed at concentrations higher than 12.5 µM in a dose-dependent manner with IC_50_ of 17.1 µM and a selectivity index (SI) of 23.4 (Figure 6B). Slight differences in IC_50_ and SI values obtained from Vero cells and porcine macrophages could be explained by structural distinctions between porcine and monkey tubulins. The time-of-addition assay demonstrated the most significant antiviral effect (2.5 log reduction, *P* < 0.05) at 8 h post-infection, which was in agreement with results in Vero cells (Figure 6C). Thus, the inhibitory effect of compound **6b** against the highly virulent ASFV strain is similar to its effect on the avirulent ASFV strain.

### Interaction with tubulin

**Figure 7.**
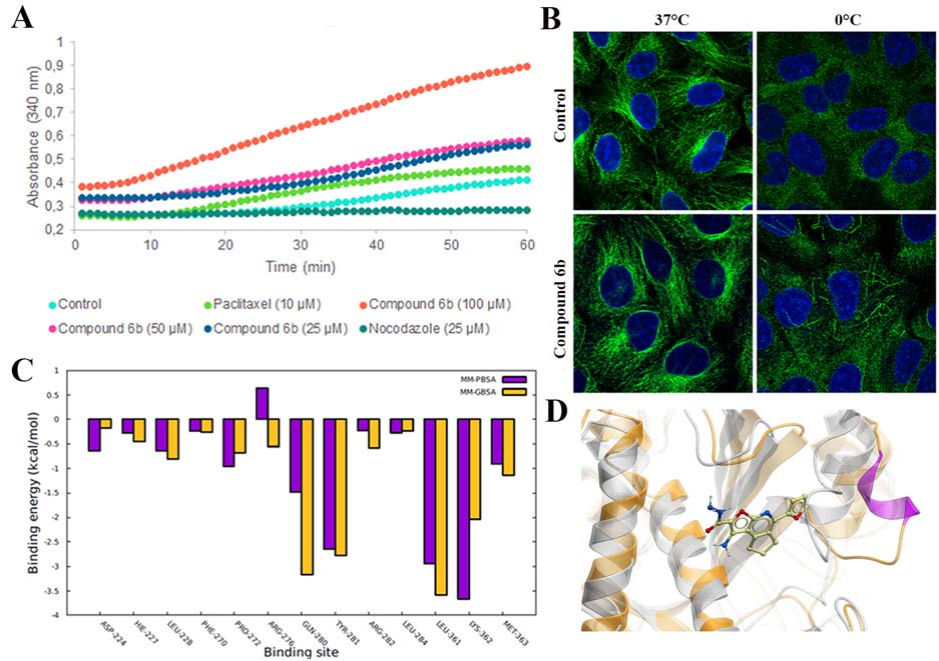
Interaction of compound **6b** with tubulin. (A) *In vitro* tubulin polymerization assay conducted with pure porcine tubulin (>99%) in the presence of compound **6b** and microtubule-targeting agents. (B) Stabilization of the tubulin filaments in Vero cells exposed to the compound 6b at 37°C and 0°C. (C) Tubulin-**6b** binding energy decomposition per amino acid in the taxane site. (D) Compound 6b in the taxane site. The grey coloured structure is the initial structure and the orange is the structure after MD simulation.

Since compound **6b** was discovered by using a virtual screening approach, we studied its biological effect on tubulin polymerization. For this purpose, porcine tubulin (>99% pure) was exposed to compound **6b** at the indicated concentrations (25–100 µM) for 1 h at 37°C. The content of polymerized tubulin was monitored by measuring the optical density every minute. At all concentrations tested, compound **6b** demonstrated progressive increase of tubulin polymerization (Figure 7A), suggesting that this compound acts as a microtubule-stabilizing rather than destabilizing agent. Then, we conducted immunofluorescence microscopy to examine the microtubule network upon treatment with compound **6b**. As shown in Figure 7B, cold treatment (15 min) resulted in the depolymerization of microtubules in mock-treated cells. In contrast, conspicuous bundles of microtubules remained in cells treated with compound **6b**, indicating that it increased the stability of microtubules in cells.

CBS ligands have proved to be potent microtubule-destabilizing agents [29,31]. Therefore, although compound **6b** was originally discovered as a CBS ligand, we supposed that it might have affinity for the target binding sites of microtubule-stabilizing agents. We investigated taxol, and laulimalide/peloruside binding pockets, known as sites for microtubule-stabilizing agents. A 4D docking study demonstrated that compound **6b** significantly bound to the taxane site (ICM score −37; MM/GBSA is −33 kcal/mol), whereas the binding score of compound **6b** to the laulimalide site was insufficient for interaction (ICM score −17; MM/GBSA is −21 kcal/mol). Next, we performed molecular dynamics (MD) simulations of compound **6b** interacting with the taxane binding site using our docked protein–ligand complex as the starting structure (supplementary video). According to our calculations, the tubulin-**6b** complex binding free energy was about −33 kcal/mol. Several amino acids had a major contribution in the binding energy, namely Gln280, Tyr281, Leu361, Lys362 (Figure 7C). Remarkably, the compound **6b** maintained the M-loop in the alpha-helical condition, which is important for tubulin lattice stabilization (Movie S1) (Figure 7D) [32].

### In vivo toxicity

To better understand the toxicity of compound **6b**, it was administered intraperitoneal to white mice at different doses up to 100 mg/kg (by a single shot). No treatment-related side effects were observed. Since the predicted half-life of compound **6b** is 1.42 h, all animals were sacrificed by day 25 to observe whether this compound or its metabolites have long-term toxic effects or not. H&E-stained sections of the liver showed a general preservation of the fundamental lobular structure. Although, some hydropic degeneration at the peripheral part of the lobule was observed indicating that the metabolic breakdown of the compound **6b** occurred in the liver (Figure S3A). The hepatocytes had intact cytoplasm, sinusoidal spaces, and prominent nuclei (Figure S3B). An increase in the number of binuclear cells was also observed (Figure S3C).

The histological analysis of kidney and bone marrow demonstrated no obvious pathological alterations (data not shown). Finally, peripheral blood analysis did not reveal significant differences between control and experimental groups, except metamyelocytes, band, and segmented neutrophils that were higher in compound **6b**-treated mice compared with control animals (Table S2). In conclusion, white mice showed a good tolerance to this compound at doses up to 100 mg/kg.

## Discussion

Due to a dense cytosolic environment where the free movement of macromolecules is restricted, microtubule-based transport is essential for virus replication. It has been shown that many human and non-human targeted viruses hijack the microtubule system in order to enter and reach specific subcellular sites for replication [33]. For example, microtubule depolymerization disrupts the trafficking of Semliki Forest virus and adeno-associated virus type 2 from early endosomes to the specific site of replication [34,35]. Other viruses such as the vaccinia and hepatitis B virus have been reported to cause microtubule-dependent recruitment of mitochondria to the sites of virus replication, thereby redirecting energy supplies for viral demand [36,37]. Finally, some viruses utilize microtubules for the proper release from the infected cells [33]. For instance, in lymphocytes, polarization of microtubules facilitates HIV-1 cell-to-cell spread through virological synapses [38,39].

Given the role of microtubules in virus replication, it is not surprising that microtubule-targeting agents can be engaged to combat viral diseases. It has been shown that colchicine, combretastatin, and their derivatives are able to inhibit HIV, dengue and Zika viruses in cell cultures at micromolar concentrations [40,41]. However, the main pharmacological disadvantage of microtubule-targeting agents regarding their use in antiviral therapy is their high cytotoxicity. Given the challenges of overcoming cytotoxicity, there is a significant opportunity to design less toxic derivatives then colchicine and combretastatin [40,41].

The emergence of the ASFV in Europe and Asia, prompts the development of effective antiviral therapies to have a significantly beneficial effect on the current situation. Potent and safe antiviral drugs will provide immediate protection in the case of an outbreak, thereby helping to contain the epidemic area and allow authorities more time to produce other countermeasures. Therefore, much effort has been made to discover and study antiviral agents against ASFV [42–47]. However, none of the described compounds have been tested *in vivo* yet, thereby highlighting the need to search for new antiviral agents targeted towards the ASFV.

Mounting evidence points to the role of microtubules in ASFV replication cycle, such as viral entry, intracellular transport, viral factory formation and egress [5–8]. Thus, we hypothesized that microtubule-targeting agents might be suitable as anti-ASFV agents. Here we showed that colchicine, nocodazole, paclitaxcel, and vinblastine could inhibit ASFV infection in Vero cells at micromolar concentrations. However, these compounds became highly toxic to cells over long treatment periods, thereby limiting their application as anti-ASFV agents and highlighting the need to find new microtubule-targeting agents that retain their biological activity but have a significant reduction of cytotoxicity.

*In vitro* screening revealed compound **6b** to be a potent inhibitor of ASFV replication in Vero cells and porcine macrophages with no evidence of cytotoxicity up to 100 µM. This compound displayed its antiviral activity at all steps of ASFV replication where microtubules were critically involved. Although the predicted half-life of compound **6b** is relatively short, time-of-removal experiments revealed that 8 h of treatment was the optimal treatment duration to achieve the strongest antiviral effect. Then, it inhibited the viral protein synthesis at different MOI, suggesting that its inhibitory effect does not depend on the viral load. Furthermore, compound **6b** suppressed the attachment of ASFV to the cell surface, thereby questioning the role of microtubules in virus attachment. Since microtubules can regulate the organization of surface receptors [48] and membrane lipid rafts [49], we assume that compound **6b** may affect these or other processes that are relevant for viral attachment to the host cell. We recommend further research to determine the mechanisms between microtubules and viral attachment.

While compound **6b** was discovered as a CBS ligand, we were intrigued by the fact that it acted as a microtubule stabilizer rather than a polymerization inhibitor. Since there is no such precedent for CBS ligands, we supposed that compound **6b** could bind to taxol or laulimalide binding pockets, known as sites for microtubule-stabilizing agents. Indeed, the 4D docking experiments and MD simulations revealed that this compound could interact with the taxane site in the same manner as many other microtubules stabilizing agents [50]. During the MD simulations, we noticed some recurrent fluctuations of the ligand in the binding site. This instability of the protein–ligand complex indicates that the binding of compound **6b** to the taxane site can be transient. This observation, as well as a relatively short biological half-life can partly explain the low toxic effects exerted by compound **6b**.

For many decades, microtubules have been considered as therapeutic targets mainly in cancer research [51,52]. Today, a large number of microtubule-targeting agents are studied as antihelmintics, fungicides and herbicides, thereby providing new therapeutic values to microtubules [53,54]. For the first time, we showed that microtubule-targeting agents could hamper ASFV infection *in vitro*. We reported about a new microtubule stabilizing agent that exhibited high biological but low cytotoxic activity. Therefore, it may have favourable properties to qualify as a new anti-ASFV parent compound that can be further modified to produce analogues with better therapeutic efficacy. Finally, this work highlights microtubules as druggable targets for the ASFV.

## Supplementary Material

s1video.mp4

S3figure.tif

S2figure.tif

S1figure.tif

Clean_copy_of_Supplementary_R2.docx
