# Supplementary figures and images for: A new microtubule-stabilizing agent shows potent antiviral effects against African swine fever virus with no cytotoxicity
Source: Emerg Microbes Infect. 2021 Mar 12;10(1):783–96. doi: 10.1080/22221751.2021.1902751 (PMC8079068; doi:10.1080/22221751.2021.1902751)

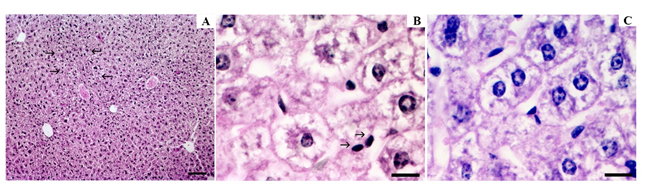

Supplement: S3figure.tif [file TEMI_A_1902751_SM6933.tif]

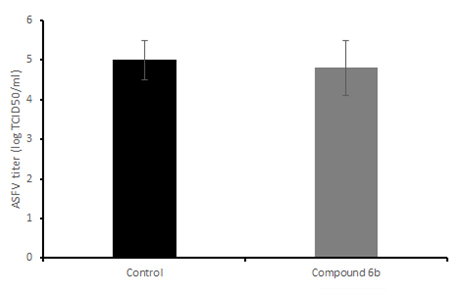

Supplement: S2figure.tif [file TEMI_A_1902751_SM6932.tif]

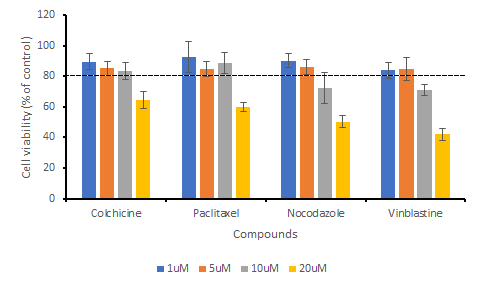

Supplement: S1figure.tif [file TEMI_A_1902751_SM6931.tif]
