# Supplementary material for: A new microtubule-stabilizing agent shows potent antiviral effects against African swine fever virus with no cytotoxicity
Source: Emerg Microbes Infect. 2021 Mar 12;10(1):783–96. doi: 10.1080/22221751.2021.1902751 (PMC8079068; doi:10.1080/22221751.2021.1902751)
Supplement: Clean_copy_of_Supplementary_R2.docx [file TEMI_A_1902751_SM6930.docx]

**Appendix**

***Material and Methods***

*Chemistry*

As starting compounds 1-aryl-3-hydroxy-6,7-dihydro-5*H*-cyclopenta[*c*]pyridine-4-carbonitriles **1a**,**b** and 1-alkyl(aryl)-3-hydroxy-5,6,7,8-tetrahydroisoquinoline-4-carbonitriles **1c**−**e** were used. Compounds **1** by simple interaction with corresponding alkylated agents [ethyl chloroacetate and 2-chloro-1-phenylethanone] in basic conditions gave the relevant *O*-alkylated derivatives **2a**−**f**. After, by the treatment of obtained ethyl [(4-cyano-1-phenyl(furyl)-6,7-dihydro-5*H*-cyclopenta[*c*]pyridin-3-yl)oxy]acetates **2a**,**b** and ethyl [(4-cyano-1-phenyl-5,6,7,8-tetrahydroisoquinolin-3-yl)oxy]acetate **2d** with potassium hydroxide in ethanol and then with hydrochloric acid the relevant [(4-cyano-1-phenyl(furyl)-6,7-dihydro-5*H*-cyclopenta[*c*]pyridin-3-yl)oxy]acetic acids **3a**,**b** and [(4-cyano-1-phenyl-5,6,7,8-tetrahydroisoquinolin-3-yl)oxy]acetic acid **3c** were synthesized (Scheme).

Further, compounds **2a−c**,**e**,**f** under the action of sodium ethylate were cyclized giving the fused furo[2,3-*b*]pyridines **5a−e**. For the synthesis of thieno[2,3-*b*]pyridine **5f**, compound **1a** were chlorinated under the action of phosphorus oxychloride. Then, the reaction of obtained 3-chloro-1-phenyl-6,7-dihydro-5*H*-cyclopenta[*c*]pyridine-4-carbonitrile **4** with ethyl mercaptoacetate in the presence of sodium ethylate led to the formation of thieno[2,3-*b*]pyridine **5f** (Scheme).

Finally, ethyl 1-aminofuro(thieno)[2,3-*b*]pyridine-2-carboxylates **5a**−**d**,**f** were reacted with hydrazine hydrate and pyrrolidine in ethanol to afford the corresponding 1-aminofuro(thieno)[2,3-*b*]pyridine-2-carbohydrazides **6a−d** and 2-(pyrrolidin-1-ylcarbonyl)-6,7,8,9-tetrahydrofuro[2,3-*c*]isoquinoline **6e**, respectively (Scheme).

The structure of all new synthesized compounds has been unambiguously confirmed by using a wide spectrum of physico-chemical methods of analysis.

**1.** **a**: R = Ph, n = 1; **b**: R = 2-furyl, n = 1; **c**: R = Me, n = 2; **d**: R = Ph, n = 2; **e**: R = 2-furyl, n = 2; **2.** **a−e**. R^1^ = OEt, **a**: R = Ph, n = 1; **b**: R = 2-furyl, n = 1; **c**: R = Me, n = 2; **d**: R = Ph, n = 2; **e**: R = 2-furyl, n = 2; **f**: R = 2-furyl, n = 1, R^1^ = Ph; **3.** **a**: R = Ph, n = 1; **b**: R = 2-furyl, n = 1; **c**: R = Ph, n = 2; **5.** **a−d**,**f.** R^1^ = OEt, **a−e.** X = O, **a**: R = Ph, n = 1; **b**: R = 2-furyl, n = 1; **c**: R = Me, n = 2; **d**: R = 2-furyl, n = 2; **e**: R = 2-furyl, n = 1, R^1^ = Ph; **f**: R = Ph, n = 1, X = S; **6.** **a**−**c**. X = O, R^2^ = NHNH_2_, **a**: R = Ph, n = 1; **b**: R = 2-furyl, n = 1; **c**: R = Me, n = 2; **d**: R = Ph, n = 1, X = S, R^2^ = NHNH_2_; **e**: R = 2-furyl, n = 2, X = O, R^2^ = pyrrolidyl.

**Scheme.** Synthesis of target compounds **3**, **5** and **6**.

*Chemistry: Experimental section*

^1^H and ^13^C NMR spectra were recorded in DMSO/CCl_4_ (1/3) solution (300 MHz for ^1^H and 75 MHz for ^13^C, respectively) on a Varian Mercury 300VX spectrometer. Chemical shifts were reported as *δ* (parts per million) relative to TMS as internal standard. IR spectra were recorded on Nicolet Avatar 330-FT-IR spectrophotometer and the reported wave numbers were given in cm^−1^. All melting points were determined in an open capillary and were uncorrected. Elemental analyses were performed on an Elemental Analyzer Euro EA 3000. Compounds **1a−e**, **2a−c**,**e** and **5a−d** were already described [22-26].

*Chemistry: General procedure for the synthesis of compounds 2d,f*

To a suspension of compound **1** (5 mmol) and potassium carbonate (76 mg, 5.5 mmol) in absolute DMF (50 mL) the corresponding alkylating agent (5.5 mmol) was added dropwise under stirring. The reaction mixture was maintained at 75**–**80 ^o^C for 2 h, then cooled to room temperature, and poured onto ice. The resulting crystals were filtered off, washed with water, dried and recrystallized from ethanol.

*Ethyl [(4-cyano-1-phenyl-5,6,7,8-tetrahydroisoquinolin-3-yl)oxy]acetate (2d). Colorless solid,* yield 81 %, mp 132‒133 ^o^C. ^1^H NMR (300 MHz, DMSO/CCl_4_, 1/3) *δ* 1.21 (t, *J* = 7.1 Hz, 3H, CH_2_CH_3_), 1.64‒1.94 (m, 4H, 6,7-CH_2_), 2.70 (t, *J* = 6.3 Hz, 2H, 8-CH_2_), 3.01 (t, *J* = 6.5 Hz, 2H, 5-CH_2_), 4.16 (q, *J* = 7.1 Hz, 2H, CH_2_CH_3_), 4.91 (s, 2H, OCH_2_), 7.36‒7.47 (m, 5H, Ph). Anal. Calcd. for C_20_H_20_N_2_O_3_: C 71.41; H 5.99; N 8.33 %. Found: C 71.74; H 6.18; N 8.57 %.

*1-(2-Furyl)-3-(2-oxo-2-phenylethoxy)-6,7-dihydro-5H-cyclopenta[c]pyridine-4-carbonitrile* **(2f).** Colorless solid, yield 73 %, mp 153−155 ^o^C. ^1^H NMR (300 MHz, DMSO/CCl_4_, 1/3) *δ* 2.19−2.35 (m, 2H, 6-CH_2_), 3.18 (t, *J* = 7.4 Hz, 2H, 7-CH_2_), 3.25 (t, *J* = 7.5 Hz, 2H, 5-CH_2_), 5.51 (s, 2H, OCH_2_), 6.49 (dd, *J* = 3.6, 1.7 Hz, 1Н, 4-CH_furyl_), 7.00 (d, *J* = 3.6 Hz, 1Н, 3-CH_furyl_), 7.35−7.43 (m, 3H, Ph), 7.57 (d, *J* = 1.7 Hz, 1Н, 5-CH_furyl_), 7.95−8.02 (m, 2H, Ph). Anal. Calcd. for C_21_H_16_N_2_O_3_: C 73.24; H 4.68; N 8.13 %. Found: C 73.62; H 4.91; N 8.40 %.

*General procedure for the synthesis of compounds 3a−c*

A mixture of compound **2a**,**b**,**d** (2 mmol) and potassium hydroxide (0.56 g, 10 mmol) in ethanol (80%, 25 mL) was refluxed for 1 h. Then the reaction mixture was neutralized with a 20% hydrochloric acid solution. The formed crystals were filtered off, washed with water, ethanol and dried.

*[(4-Cyano-1-phenyl-6,7-dihydro-5H-cyclopenta[c]pyridin-3-yl)oxy]acetic acid (3a).*

Colorless solid, yield 77 %, mp 205–207 ^o^C; IR *ν*/cm^−1^: 1738 (C=О), 2223 (C≡N). ^1^H NMR (300 MHz, DMSO/CCl_4_, 1/3) *δ* 2.17−2.27 (m, 2H, 6-CH_2_), 3.14 (t, *J* = 7.3 Hz, 2H, 7-CH_2_), 3.16 (t, *J* = 7.5 Hz, 2H, 5-CH_2_), 3.21 (br, 1H, OH), 5.05 (s, 2H, OCH_2_), 7.40–7.45 (m, 3H, Ph), 7.71–7.75 (m, 2H, Ph). Anal. Calcd. for C_17_H_14_N_2_O_3_: C 69.38; H 4.79; N 9.52 %. Found: C 69.72; H 4.99; N 9.75 %.

*[(4-Cyano-1-(2-furyl)-6,7-dihydro-5H-cyclopenta[c]pyridin-3-yl]oxy]acetic acid (3b).* Colorless solid, yield 74 %, mp 248‒250 ^o^C. ^1^H NMR (300 MHz, DMSO/CCl_4_, 1/3) *δ* 2.19‒2.30 (m, 2H, 6-CH_2_), 3.09 (t, *J* = 7.7 Hz, 2H, 7-CH_2_), 3.25 (t, *J* = 7.5 Hz, 2H, 5-CH_2_), 3.22 (br, 1H, OH), 4.88 (s, 2H, OCH_2_), 6.61 (dd, *J* = 3.5, 1.8 Hz, 1H, 4-CH_furyl_), 7.12 (dd, *J* = 3.5, 0.8 Hz, 1H, 3-CH_furyl_), 7.71 (dd, *J* = 1.8, 0.8 Hz, 1H, 5-CH_furyl_). Anal. Calcd. for C_15_H_12_N_2_O_4_ C: 63.38; H 4.25; N 9.85 %. Found: 63.75; 4.48; N 10.11 %.

*[(4-Cyano-1-phenyl-5,6,7,8-tetrahydroisoquinolin-3-yl)oxy]acetic acid (3c). Colorless solid,* yield 81 %, mp 162‒164 ^o^C. ^1^H NMR (300 MHz, DMSO/CCl_4_, 1/3) *δ* 1.64‒1.94 (m, 4H, 6,7-CH_2_), 2.70 (t, *J* = 6.3 Hz, 2H, 8-CH_2_), 3.01 (t, *J* = 6.5 Hz, 2H, 5-CH_2_), 3.15 (br, 1H, OH), 4.87 (s, 2H, OCH_2_), 7.36‒7.51 (m, 5H, Ph). Anal. Calcd. for C_18_H_16_N_2_O_3_: C 70.12; H 5.23; N 9.09 %. Found: C 70.47; H 5.44; N 9.33 %.

*Procedure for the synthesis of compound 4*

A mixture of compound **1a** (2.36 g, 10 mmol) and phosphorus oxychloride (50 mL) was refluxed on a water bath for 5 h. After, the excess of solvent was distilled off to dryness, and ice water was added to the residue. Then the reaction mixture was neutralized with an aqueous solution of potassium hydroxide (10 %) under stirring. The separated crystals were filtered off, washed with water, dried, and recrystallized from ethanol.

*3-Chloro-1-phenyl-6,7-dihydro-5H-cyclopenta[c]pyridine-4-carbonitrile (4). Colorless solid,* yield 78 %, mp 151‒153 ^o^C; IR *ν*/cm^−1^: 2223 (C≡N). ^1^H NMR (300 MHz, DMSO/CCl_4_, 1/3) *δ* 2.18–2.29 (m, 2H, 6-CH_2_), 3.18 (t, *J* = 7.6 Hz, 2H, 7-CH_2_), 3.22 (t, *J* = 7.4 Hz, 2H, 5-CH_2_), 7.45–7.53 (m, 3H, Ph), 7.80–7.86 (m, 2H, Ph). Anal. Calcd. for C_15_H_11_ClN_2_: C 70.73; H 4.35; N 11.00 %. Found: C 71.11; H 4.54; N 11.27 %.

*Procedure for the synthesis of compound 5e*

To a solution of sodium ethylate [0.13 g (5.5 mmol) of sodium in absolute ethanol (50 mL)] compound **2f** (1.72 g, 5 mmol) was added. The mixture was refluxed for 2 h, cooled, and poured onto water. The formed crystals were filtered off, washed with water, dried and recrystallized from ethanol.

*[1-Amino-5-(2-furyl)-7,8-dihydro-6H-cyclopenta[d]furo[2,3-b]pyridin-2-yl](phenyl)-methanone (5e).*

Light yellow solid, yield 83 %, mp 207−209 ^o^C; IR *ν*/cm^−1^: 3535, 3280 (NH_2_), 1687 (C=O). ^1^H NMR (300 MHz, DMSO/CCl_4_, 1/3) *δ* 2.22−2.38 (m, 2H, 7-CH_2_), 3.32 (t, *J* = 7.4 Hz, 2H, 6-CH_2_), 3.37 (t, *J* = 7.5 Hz, 2H, 8-CH_2_), 6.60 (dd, *J* = 3.6, 1.7 Hz, 1Н, 4-CH_furyl_), 7.00 (br s, 2H, NH_2_), 7.15 (d, *J* = 3.6 Hz, 1Н, 3-CH_furyl_), 7.48−7.56 (m, 3H, Ph), 7.68 (d, *J* = 1.7 Hz, 1Н, 5-CH_furyl_), 8.16−8.23 (m, 2H, Ph). Anal. Calcd. for C_21_H_16_N_2_O_3_: C 73.24; H 4.68; N 8.13 %. Found: C 73.63; H 4.92; N 8.41 %.

*Procedure for the synthesis of compound 5f*

To a solution of sodium ethylate [0.14 g (6 mmol) of sodium in absolute ethanol (50 mL)], ethyl 2-mercaptoacetate (0.72 g, 6 mmol) and compound **4** (1.27 g, 5 mmol) were added. The mixture was refluxed for 5 h, cooled, and poured onto ice water. The formed crystals were filtered off, washed with water, dried, and recrystallized from ethanol.

*Ethyl 1-amino-5-phenyl-7,8-dihydro-6H-cyclopenta[d]thieno[2,3-b]pyridine-2-carboxylate* **(5f)**. Light yellow solid, yield 80 %, mp 179–181 ^o^C; IR *ν*/cm^−1^: 1609 (C=O), 3456, 3342 (NH_2_). ^1^H NMR (300 MHz, DMSO/CCl_4_, 1/3) *δ* 1.40 (t, *J* = 7.1 Hz, 3H, CH_2_CH_3_), 2.15−2.27 (m, 2H, 7-CH_2_), 3.15 (t, *J* = 7.5 Hz, 2Н, 6-CH_2_), 3.45 (t, *J* = 7.5 Hz, 2H, 8-CH_2_), 4.31 (q, *J* = 7.1 Hz, 2H, CH_2_CH_3_), 6.52 (br s, 2H, NH_2_), 7.35−7.48 (m, 3H, Ph), 7.77−7.81 (m, 2H, Ph). Anal. Calcd. for C_19_H_18_N_2_O_2_S: C 67.43; H 5.36; N 8.28 %. Found: C 67.77; H 5.54; N 8.51 %.

*General procedure for the synthesis of compounds 6a−e*

A mixture of compound **5a**−**d**,**f** (2 mmol) and hydrazine hydrate (1 g, 20 mmol) [or pyrrolidine (0.18 mL, 2.2 mmol) for compound **6e**] in absolute ethanol (25 mL) was refluxed for 10 h. The reaction mixture was cooled, water (50 mL) was added. The separated crystals were filtered off, washed with water, dried, and recrystallized from ethanol.

*1-Amino-5-phenyl-7,8-dihydro-6H-cyclopenta[d]furo[2,3-b]pyridine-2-carbohydrazide (6a).* Light yellow solid, yield 74 %, mp 228−230 ^o^C; IR *ν*/cm^−1^: 1634 (C=O), 3253, 3349, 3437 (NH, NH_2_). ^1^H NMR (300 MHz, DMSO/CCl_4_, 1/3) *δ* 2.22 (m, 2H, 7-CH_2_), 3.14 (t, *J* = 7.3 Hz, 2H, 6-CH_2_), 3.35 (t, *J* = 7.5 Hz, 2H, 8-CH_2_), 4.20 (br s, 2H, NHNH_2_), 5.57 (br s, 2H, NH_2_), 7.33‒7.54 (m, 3H, Ph), 7.70‒7.79 (m, 2H, Ph), 9.05 (br, 1H, NH). ^13^C NMR (75 MHz, DMSO/CCl_4_, 1/3) δ 25.62, 30.51, 31.56, 110.66, 125.20, 127.52, 127.63, 128.04, 131.89, 134.30, 139.34, 149.80, 150.37, 158.13, 160.73. Anal. Calcd. for C_17_H_16_N_4_O_2_: C 66.22; H 5.23; N 18.17 %. Found: C 66.59; H 5.45; N 18.43 %.

*1-Amino-5-(2-furyl)-7,8-dihydro-6H-cyclopenta[d]furo[2,3-b]pyridine-2-carbohydrazide* **(6b).** Light yellow solid, yield 83 %, mp 273−275 ^o^C; IR *ν*/cm^−1^: 1620 (C=O); 3318, 3345 3437 (NH, NH_2_). ^1^H NMR (300 MHz, DMSO/CCl_4_, 1/3) *δ* 2.21 (m, 2H, 7-CH_2_), 3.19 (t, *J* = 7.4 Hz, 2H, 6-CH_2_), 3.30 (t, *J* = 7.5 Hz, 2H, 8-CH_2_), 4.37 (br, 2H, NHNH_2_), 5.74 (br s, 2H, NH_2_), 6.68 (dd, *J* = 3.6, 1.7 Hz, 1Н, 4-CH_furyl_), 7.06 (d, *J* = 3.6 Hz, 1Н, 3-CH_furyl_), 7.88 (d, *J* = 1.7 Hz, 1Н, 5-CH_furyl_), 9.32 (br, 1H, NH). ^13^C NMR (75 MHz, DMSO/CCl_4_, 1/3) δ 24.66, 30.38, 30.94, 110.57, 110.90, 112.15, 125.27, 130.38, 134.42, 141.17, 144.37, 150.76, 156.12, 157.72, 160.81. Anal. Calcd. for C_15_H_14_N_4_O_3_: C 60.40; H 4.73; N 18.78 %. Found: C 60.71; H 4.93; N 19.02 %.

*1-Amino-5-methyl-6,7,8,9-tetrahydrofuro[2,3-c]isoquinoline-2-carbohydrazide (6c).*

Light yellow solid, yield 75 %, mp 270−272 ^o^C; IR *ν*/cm^−1^: 1613 (C=O), 3283 (NH, NH_2_). ^1^H NMR (300 MHz, DMSO/CCl_4_, 1/3) *δ* 1.77–1.91 (m, 4H, 7,8-CH_2_), 2.42 (s, 3H, CH_3_), 2.62–2.67 (m, 2H, 6-CH_2_), 3.15–3.20 (m, 2H, 9-CH_2_), 4.20 (br, 2H, NHNH_2_), 5.52 (br s, 2H, NH_2_), 8.97 (br, 1H, NH). Anal. Calcd. for C_13_H_16_N_4_O_2_: C 59.99; H 6.20; N 21.52 %. Found: C 60.34; H 6.38; N 21.75 %.

*1-Amino-5-phenyl-7,8-dihydro-6H-cyclopenta[d]thieno[2,3-b]pyridine-2-carbohydrazide* **(6d).** Light yellow solid, yield 77 %, mp 220−222 ^o^C; IR *ν*/cm^−1^: 1630 (C=O), 3247, 3364, 3451 (NH, NH_2_). ^1^H NMR (300 MHz, DMSO/CCl_4_, 1/3) *δ* 2.18 (m, 2H, 7-CH_2_), 3.12 (t, *J* = 7.5 Hz, 2H, 6-CH_2_), 3.44 (t, *J* = 7.6 Hz, 2H, 8-CH_2_), 4.40 (br, 3H, NHNH_2_), 6.49 (br s, 2H, NH_2_), 7.31‒7.48 (m, 3H, Ph), 7.70‒7.82 (m, 2H, Ph). ^13^C NMR (75 MHz, DMSO/CCl_4_, 1/3) δ 24.88, 31.14, 31.61, 97.67, 122.36, 127.46, 127.79, 128.16, 132.91, 139.21, 145.73, 149.56, 153.35, 157.22, 165.35. Anal. Calcd. for C_17_H_16_N_4_OS: C 62.94; H 4.97; N 17.27 %. Found: C 63.27; H 5.18; N 17.52 %.

*5-(2-Furyl)-2-(pyrrolidin-1-ylcarbonyl)-6,7,8,9-tetrahydrofuro[2,3-c]isoquinolin-1-amine* *(6e).* Light yellow solid, yield 85 %, mp 228−230 ^o^C; IR *ν*/cm^−1^: 1643 (C=O), 3434, 3342 (NН_2_). ^1^H NMR (300 MHz, DMSO/CCl_4_, 1/3) *δ* 1.75–2.15 (m, 8H, 7,8-CH_2_, 2CH_2_-C_4_H_8_N), 3.00–3.10 (m, 2H, 6-CH_2_), 3.24–3.33 (m, 2H, 9-CH_2_), 3.38−4.00 (m, 4H, N(CH_2_)_2_), 5.78 (br s, 2H, NH_2_), 6.54 (dd, *J* = 3.5, 1.8 Hz, 1H, 4-CH_furyl_), 6.96 (dd, *J* = 3.5, 0.8 Hz, 1H, 3-CH_furyl_), 7.62 (dd, *J* = 1.8, 0.8 Hz, 1H, 5-CH_furyl_). Anal. Calcd. for C_20_H_21_N_3_O_3_: C 68.36; H 6.02; N 11.96 %. Found: C 68.72; H 6.19; N 12.18 %.

*Confocal microscopy*

Vero cells seeded on glass coverslips (1 × 10^5^/cm^2^), either mock-infected or infected with the ASFV Ba71V (1 TCID50/cell), were exposed to the compound (100 µM) or to solvent alone (DMSO); exposure to drug or solvent was for either 8 or 16 hours before collection. At these time points, cells were fixed in 3.7% paraformaldehyde in HPEM buffer [30 mM HEPES (4-(2-hydroxyethyl)-1-piperazineethanesulfonic acid) containing 65 mM PIPES (piperazine-N,N′-bis 2-ethanesulfonic acid), 10 mM EGTA (ethylene glycol tetraacetic acid) and 2 mM MgCl_2_; pH 6.9] plus 0.5% (v/v) Triton X-100 for 10 min, at room temperature, before incubation with antibodies. All washes were done with PBS containing 0.05% Triton X-100, and antibodies were diluted in PBS containing fetal bovine serum (5% v/v; blocking agent), 0.05% (v/v) Triton X-100 and 0.1% (w/v) sodium azide.

The immunostaining of ASFV-pI215L and ASFV-infected cells were achieved by incubation with two in-house primary antibodies: mouse anti-ASFV-PI215L and anti-ASFV swine serum directly conjugated to fluorescein isothiocyanate (FITC; 1:100, 1 h, RT) as previously described [47, 48]. The affinity purified Alexa Fluor 568-conjugated goat anti-mouse secondary antibody (Thermo Fisher Scientific; A-11031; 1:150) was used to detect the ASFV-pI215L, whereas the monoclonal rat anti-tubulin antibody (Abcam; ab 6160; 1:1000) and the Alexa Fluor 488-conjugated donkey anti-rat (Thermo Fisher Scientific; 1:150; A-21208) were used to evaluate the effects of the compound 6b on the tubulin network. For double-immunostaining experiments primary antibodies were mixed to the specified final concentrations. Between each antibody incubation, cells were washed twice with PBS for 5 minutes and once with PBST (0.1% v/v for 5 minutes). All incubations were performed in a dark humidified chamber to prevent fluorochrome fading. After immunolabeling, cell nucleus and viral factories were stained with DAPI (4′,6-diamidino-2-phenylindole, 0.5 μg/mL) and coverslips were mounted in Vectashield (Vector Laboratories Inc., Burlingame, CA, USA) before analysis by fluorescence microscopy.

In some experiments, ASFV-infected cells were exposed to EdU (5-ethynyl-2′-deoxyuridine; 25 microM) for 15 min before collection; cells exposed to solvent (DMSO) served as controls. EdU incorporated into replicating DNA was detected with Click-iT EdU 6-FAM azide kit following the manufacturer's instructions (Baseclick GmbH, Neuried, Germany).

Samples were examined using a Zeiss 710 confocal microscope (Carl Zeiss, Jena, Germany) equipped with lasers giving excitation lines at 405, 488 and 561 nm. Data from the channels were collected separately using narrow-band-pass filter settings. In multiple staining experiments, the laser intensities were adjusted to avoid bleed-through between channels. Data was collected with two- to four-fold averaging at resolution of 1024 × 1024 pixels using pinhole settings between 1.05 and 1.10 airy units. Data sets were processed using Zeiss 710 version 2.8 software package and were subsequently exported for preparation for printing using Adobe Photoshop, version CS5.1 (Adobe Systems, Inc., San Jose, USA).

*Histology and blood cells analysis*

All animals (white mice; both sexes; 20-25g; n_control_ = 5; n_exp_ = 4 for each dose) were sacrificed at 25 days post-administration according to the institutional and national guidelines for laboratory animals. Tissue samples from liver and spleen were fixed in 10 % buffered formalin (pH 7.2) for 24 hours. After fixation, samples were dehydrated through a graded series of alcohols, washed with xylol and embedded in paraffin wax by a routine technique for light microscopy. For anatomopathological examination, wax-embedded samples were cut (Microm HM 355, 5 µm) and stained with hematoxylin and eosin according to the manufacturer’s protocol (Sigma-Aldrich, Germany). The histological examination was carried out using a light microscope.

All blood samples were collected from the jugular vein of mice. Fresh blood was used in preparing the blood smears by routine methods. For nucleated blood cells analysis, slides were fixed in pure methanol and stained by Giemsa modified solution (azure B/azure II, eosin and methylene blue) according to the manufacturer’s protocol (Sigma-Aldrich, Germany), Slides were fixed for 10 minutes in methanol, immersed for 45 minutes in Giemsa solution and rinsed with distilled water. White blood cells were examined under the light microscope at ×1250 in a random sequence. At least 200 white blood cells in each sample were evaluated for cell types.

**Results**

**
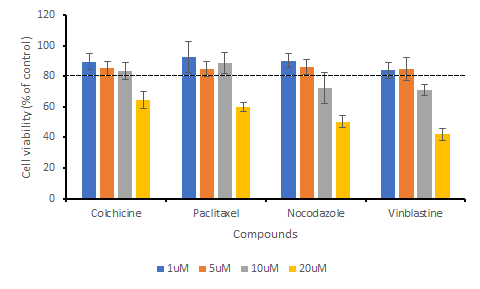
**

**Figure 1S. Cytotoxicity of microtubule-targeting agents on Vero cells**. The cytotoxicity of drugs was evaluated by MTT assay at 24 h of incubation. Drug concentrations were considered non-cytotoxic if cell viability was higher than 80% of control (dash line).

**
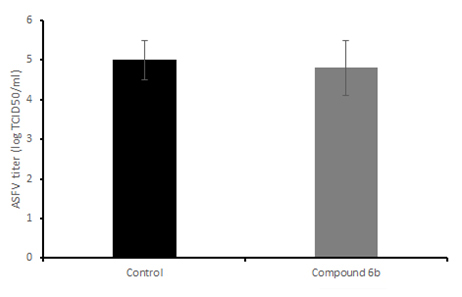
**

**Figure S2. Direct effect of compound 6b on extracellular ASFV particles.** Virus was co-incubated with compound **6b** at 100 µM concentration at room temperature. ASFV titers were determined by CPE-based assay.


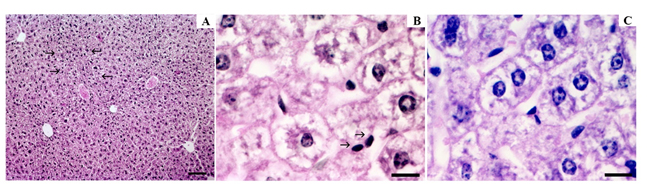


**Figure S3. Liver sections stained by H&E.** (A) Development of hydropic degeneration in hepatocytes. Hydropic degeneration in peripheral part of classic lobuli (indicated by arrows). Scale is 50µm. (B) Central localization of hepatocyte nucleus in vacuolated cells. Scale is 10µm. (C) Binuclear hepatocytes. Scale 10µm.

**Table S1. The half-life of compound 6b and tubulin-targeting drugs predicted by ICM software.**

| **Compound** | **PubChem ID** | **Plasma Half-life (hours)** |
| --- | --- | --- |
| Compound 6b | - | 1.425146 |
| Colchicine | 6167 | 6.024944 |
| Paclitaxel | 36314 | 3.32472 |
| Nocodazole | 4122 | 2.471672 |
| Vinblastine | 13342 | 1.914483 |

**Table S2. Composition of blood cells.**

| **Cell types** | **The percent (%) of cells** | |
| --- | --- | --- |
|  | **Control (mean** ± SD) | **Compound 6b (mean** ± SD) |
| Erythroblasts | 29.5 ± 6.1 | 26.2 ± 5.1 |
| Lymphoblasts | 6.3 ± 1.1 | 4.9 ± 1.0 |
| Lymphocytes | 28.4 ± 5.4 | 23.2 ± 4.9 |
| Monoblasts | 2.1 ± 1.0 | 3.5 ± 1.7 |
| Monocytes | 2.7 ± 1.0 | 2.6 ± 1.1 |
| Myeloids | 4.8 ± 0.9 | 5.3 ± 1.6 |
| Metamyelocytes | 3.6 ± 1.8 | 6.3 ± 1.1* |
| Band neutrophils | 9.1 ± 2.2 | 12.8 ± 2.7* |
| Segmented neutrophils | 9.5 ± 2.7 | 11.4 ± 2.6* |
| Eosinophils | 3.3 ± 1.4 | 3.2 ± 1.2 |
| Basophils | 0.2 ± 0.1 | 0.1 ± 0.1 |
| Megakaryocytes | 0.3 ± 0.2 | 0.3 ± 0.1 |
| Macrophages | 0.2 ± 0.1 | 0.2 ± 0.3 |
| Pathological forms | - | 0.1 ± 0.2 |

*significant increase compared with control (p<0.05 – p<0.01)

**S1 video. MD simulation of compound 6b in the taxane site.** The M-loop is rearranged into a short alpha-helix and changes of secondary structures are color-coded: loop - cyan, 3-10 helix - blue, alpha-helix – magenta.

**Appendix**

***Material and Methods***

*Chemistry*

As starting compounds 1-aryl-3-hydroxy-6,7-dihydro-5*H*-cyclopenta[*c*]pyridine-4-carbonitriles **1a**,**b** and 1-alkyl(aryl)-3-hydroxy-5,6,7,8-tetrahydroisoquinoline-4-carbonitriles **1c**−**e** were used. Compounds **1** by simple interaction with corresponding alkylated agents [ethyl chloroacetate and 2-chloro-1-phenylethanone] in basic conditions gave the relevant *O*-alkylated derivatives **2a**−**f**. After, by the treatment of obtained ethyl [(4-cyano-1-phenyl(furyl)-6,7-dihydro-5*H*-cyclopenta[*c*]pyridin-3-yl)oxy]acetates **2a**,**b** and ethyl [(4-cyano-1-phenyl-5,6,7,8-tetrahydroisoquinolin-3-yl)oxy]acetate **2d** with potassium hydroxide in ethanol and then with hydrochloric acid the relevant [(4-cyano-1-phenyl(furyl)-6,7-dihydro-5*H*-cyclopenta[*c*]pyridin-3-yl)oxy]acetic acids **3a**,**b** and [(4-cyano-1-phenyl-5,6,7,8-tetrahydroisoquinolin-3-yl)oxy]acetic acid **3c** were synthesized (Scheme).

Further, compounds **2a−c**,**e**,**f** under the action of sodium ethylate were cyclized giving the fused furo[2,3-*b*]pyridines **5a−e**. For the synthesis of thieno[2,3-*b*]pyridine **5f**, compound **1a** were chlorinated under the action of phosphorus oxychloride. Then, the reaction of obtained 3-chloro-1-phenyl-6,7-dihydro-5*H*-cyclopenta[*c*]pyridine-4-carbonitrile **4** with ethyl mercaptoacetate in the presence of sodium ethylate led to the formation of thieno[2,3-*b*]pyridine **5f** (Scheme).

Finally, ethyl 1-aminofuro(thieno)[2,3-*b*]pyridine-2-carboxylates **5a**−**d**,**f** were reacted with hydrazine hydrate and pyrrolidine in ethanol to afford the corresponding 1-aminofuro(thieno)[2,3-*b*]pyridine-2-carbohydrazides **6a−d** and 2-(pyrrolidin-1-ylcarbonyl)-6,7,8,9-tetrahydrofuro[2,3-*c*]isoquinoline **6e**, respectively (Scheme).

The structure of all new synthesized compounds has been unambiguously confirmed by using a wide spectrum of physico-chemical methods of analysis.

**1.** **a**: R = Ph, n = 1; **b**: R = 2-furyl, n = 1; **c**: R = Me, n = 2; **d**: R = Ph, n = 2; **e**: R = 2-furyl, n = 2; **2.** **a−e**. R^1^ = OEt, **a**: R = Ph, n = 1; **b**: R = 2-furyl, n = 1; **c**: R = Me, n = 2; **d**: R = Ph, n = 2; **e**: R = 2-furyl, n = 2; **f**: R = 2-furyl, n = 1, R^1^ = Ph; **3.** **a**: R = Ph, n = 1; **b**: R = 2-furyl, n = 1; **c**: R = Ph, n = 2; **5.** **a−d**,**f.** R^1^ = OEt, **a−e.** X = O, **a**: R = Ph, n = 1; **b**: R = 2-furyl, n = 1; **c**: R = Me, n = 2; **d**: R = 2-furyl, n = 2; **e**: R = 2-furyl, n = 1, R^1^ = Ph; **f**: R = Ph, n = 1, X = S; **6.** **a**−**c**. X = O, R^2^ = NHNH_2_, **a**: R = Ph, n = 1; **b**: R = 2-furyl, n = 1; **c**: R = Me, n = 2; **d**: R = Ph, n = 1, X = S, R^2^ = NHNH_2_; **e**: R = 2-furyl, n = 2, X = O, R^2^ = pyrrolidyl.

**Scheme.** Synthesis of target compounds **3**, **5** and **6**.

*Chemistry: Experimental section*

^1^H and ^13^C NMR spectra were recorded in DMSO/CCl_4_ (1/3) solution (300 MHz for ^1^H and 75 MHz for ^13^C, respectively) on a Varian Mercury 300VX spectrometer. Chemical shifts were reported as *δ* (parts per million) relative to TMS as internal standard. IR spectra were recorded on Nicolet Avatar 330-FT-IR spectrophotometer and the reported wave numbers were given in cm^−1^. All melting points were determined in an open capillary and were uncorrected. Elemental analyses were performed on an Elemental Analyzer Euro EA 3000. Compounds **1a−e**, **2a−c**,**e** and **5a−d** were already described [22-26].

*Chemistry: General procedure for the synthesis of compounds 2d,f*

To a suspension of compound **1** (5 mmol) and potassium carbonate (76 mg, 5.5 mmol) in absolute DMF (50 mL) the corresponding alkylating agent (5.5 mmol) was added dropwise under stirring. The reaction mixture was maintained at 75**–**80 ^o^C for 2 h, then cooled to room temperature, and poured onto ice. The resulting crystals were filtered off, washed with water, dried and recrystallized from ethanol.

*Ethyl [(4-cyano-1-phenyl-5,6,7,8-tetrahydroisoquinolin-3-yl)oxy]acetate (2d). Colorless solid,* yield 81 %, mp 132‒133 ^o^C. ^1^H NMR (300 MHz, DMSO/CCl_4_, 1/3) *δ* 1.21 (t, *J* = 7.1 Hz, 3H, CH_2_CH_3_), 1.64‒1.94 (m, 4H, 6,7-CH_2_), 2.70 (t, *J* = 6.3 Hz, 2H, 8-CH_2_), 3.01 (t, *J* = 6.5 Hz, 2H, 5-CH_2_), 4.16 (q, *J* = 7.1 Hz, 2H, CH_2_CH_3_), 4.91 (s, 2H, OCH_2_), 7.36‒7.47 (m, 5H, Ph). Anal. Calcd. for C_20_H_20_N_2_O_3_: C 71.41; H 5.99; N 8.33 %. Found: C 71.74; H 6.18; N 8.57 %.

*1-(2-Furyl)-3-(2-oxo-2-phenylethoxy)-6,7-dihydro-5H-cyclopenta[c]pyridine-4-carbonitrile* **(2f).** Colorless solid, yield 73 %, mp 153−155 ^o^C. ^1^H NMR (300 MHz, DMSO/CCl_4_, 1/3) *δ* 2.19−2.35 (m, 2H, 6-CH_2_), 3.18 (t, *J* = 7.4 Hz, 2H, 7-CH_2_), 3.25 (t, *J* = 7.5 Hz, 2H, 5-CH_2_), 5.51 (s, 2H, OCH_2_), 6.49 (dd, *J* = 3.6, 1.7 Hz, 1Н, 4-CH_furyl_), 7.00 (d, *J* = 3.6 Hz, 1Н, 3-CH_furyl_), 7.35−7.43 (m, 3H, Ph), 7.57 (d, *J* = 1.7 Hz, 1Н, 5-CH_furyl_), 7.95−8.02 (m, 2H, Ph). Anal. Calcd. for C_21_H_16_N_2_O_3_: C 73.24; H 4.68; N 8.13 %. Found: C 73.62; H 4.91; N 8.40 %.

*General procedure for the synthesis of compounds 3a−c*

A mixture of compound **2a**,**b**,**d** (2 mmol) and potassium hydroxide (0.56 g, 10 mmol) in ethanol (80%, 25 mL) was refluxed for 1 h. Then the reaction mixture was neutralized with a 20% hydrochloric acid solution. The formed crystals were filtered off, washed with water, ethanol and dried.

*[(4-Cyano-1-phenyl-6,7-dihydro-5H-cyclopenta[c]pyridin-3-yl)oxy]acetic acid (3a).*

Colorless solid, yield 77 %, mp 205–207 ^o^C; IR *ν*/cm^−1^: 1738 (C=О), 2223 (C≡N). ^1^H NMR (300 MHz, DMSO/CCl_4_, 1/3) *δ* 2.17−2.27 (m, 2H, 6-CH_2_), 3.14 (t, *J* = 7.3 Hz, 2H, 7-CH_2_), 3.16 (t, *J* = 7.5 Hz, 2H, 5-CH_2_), 3.21 (br, 1H, OH), 5.05 (s, 2H, OCH_2_), 7.40–7.45 (m, 3H, Ph), 7.71–7.75 (m, 2H, Ph). Anal. Calcd. for C_17_H_14_N_2_O_3_: C 69.38; H 4.79; N 9.52 %. Found: C 69.72; H 4.99; N 9.75 %.

*[(4-Cyano-1-(2-furyl)-6,7-dihydro-5H-cyclopenta[c]pyridin-3-yl]oxy]acetic acid (3b).* Colorless solid, yield 74 %, mp 248‒250 ^o^C. ^1^H NMR (300 MHz, DMSO/CCl_4_, 1/3) *δ* 2.19‒2.30 (m, 2H, 6-CH_2_), 3.09 (t, *J* = 7.7 Hz, 2H, 7-CH_2_), 3.25 (t, *J* = 7.5 Hz, 2H, 5-CH_2_), 3.22 (br, 1H, OH), 4.88 (s, 2H, OCH_2_), 6.61 (dd, *J* = 3.5, 1.8 Hz, 1H, 4-CH_furyl_), 7.12 (dd, *J* = 3.5, 0.8 Hz, 1H, 3-CH_furyl_), 7.71 (dd, *J* = 1.8, 0.8 Hz, 1H, 5-CH_furyl_). Anal. Calcd. for C_15_H_12_N_2_O_4_ C: 63.38; H 4.25; N 9.85 %. Found: 63.75; 4.48; N 10.11 %.

*[(4-Cyano-1-phenyl-5,6,7,8-tetrahydroisoquinolin-3-yl)oxy]acetic acid (3c). Colorless solid,* yield 81 %, mp 162‒164 ^o^C. ^1^H NMR (300 MHz, DMSO/CCl_4_, 1/3) *δ* 1.64‒1.94 (m, 4H, 6,7-CH_2_), 2.70 (t, *J* = 6.3 Hz, 2H, 8-CH_2_), 3.01 (t, *J* = 6.5 Hz, 2H, 5-CH_2_), 3.15 (br, 1H, OH), 4.87 (s, 2H, OCH_2_), 7.36‒7.51 (m, 5H, Ph). Anal. Calcd. for C_18_H_16_N_2_O_3_: C 70.12; H 5.23; N 9.09 %. Found: C 70.47; H 5.44; N 9.33 %.

*Procedure for the synthesis of compound 4*

A mixture of compound **1a** (2.36 g, 10 mmol) and phosphorus oxychloride (50 mL) was refluxed on a water bath for 5 h. After, the excess of solvent was distilled off to dryness, and ice water was added to the residue. Then the reaction mixture was neutralized with an aqueous solution of potassium hydroxide (10 %) under stirring. The separated crystals were filtered off, washed with water, dried, and recrystallized from ethanol.

*3-Chloro-1-phenyl-6,7-dihydro-5H-cyclopenta[c]pyridine-4-carbonitrile (4). Colorless solid,* yield 78 %, mp 151‒153 ^o^C; IR *ν*/cm^−1^: 2223 (C≡N). ^1^H NMR (300 MHz, DMSO/CCl_4_, 1/3) *δ* 2.18–2.29 (m, 2H, 6-CH_2_), 3.18 (t, *J* = 7.6 Hz, 2H, 7-CH_2_), 3.22 (t, *J* = 7.4 Hz, 2H, 5-CH_2_), 7.45–7.53 (m, 3H, Ph), 7.80–7.86 (m, 2H, Ph). Anal. Calcd. for C_15_H_11_ClN_2_: C 70.73; H 4.35; N 11.00 %. Found: C 71.11; H 4.54; N 11.27 %.

*Procedure for the synthesis of compound 5e*

To a solution of sodium ethylate [0.13 g (5.5 mmol) of sodium in absolute ethanol (50 mL)] compound **2f** (1.72 g, 5 mmol) was added. The mixture was refluxed for 2 h, cooled, and poured onto water. The formed crystals were filtered off, washed with water, dried and recrystallized from ethanol.

*[1-Amino-5-(2-furyl)-7,8-dihydro-6H-cyclopenta[d]furo[2,3-b]pyridin-2-yl](phenyl)-methanone (5e).*

Light yellow solid, yield 83 %, mp 207−209 ^o^C; IR *ν*/cm^−1^: 3535, 3280 (NH_2_), 1687 (C=O). ^1^H NMR (300 MHz, DMSO/CCl_4_, 1/3) *δ* 2.22−2.38 (m, 2H, 7-CH_2_), 3.32 (t, *J* = 7.4 Hz, 2H, 6-CH_2_), 3.37 (t, *J* = 7.5 Hz, 2H, 8-CH_2_), 6.60 (dd, *J* = 3.6, 1.7 Hz, 1Н, 4-CH_furyl_), 7.00 (br s, 2H, NH_2_), 7.15 (d, *J* = 3.6 Hz, 1Н, 3-CH_furyl_), 7.48−7.56 (m, 3H, Ph), 7.68 (d, *J* = 1.7 Hz, 1Н, 5-CH_furyl_), 8.16−8.23 (m, 2H, Ph). Anal. Calcd. for C_21_H_16_N_2_O_3_: C 73.24; H 4.68; N 8.13 %. Found: C 73.63; H 4.92; N 8.41 %.

*Procedure for the synthesis of compound 5f*

To a solution of sodium ethylate [0.14 g (6 mmol) of sodium in absolute ethanol (50 mL)], ethyl 2-mercaptoacetate (0.72 g, 6 mmol) and compound **4** (1.27 g, 5 mmol) were added. The mixture was refluxed for 5 h, cooled, and poured onto ice water. The formed crystals were filtered off, washed with water, dried, and recrystallized from ethanol.

*Ethyl 1-amino-5-phenyl-7,8-dihydro-6H-cyclopenta[d]thieno[2,3-b]pyridine-2-carboxylate* **(5f)**. Light yellow solid, yield 80 %, mp 179–181 ^o^C; IR *ν*/cm^−1^: 1609 (C=O), 3456, 3342 (NH_2_). ^1^H NMR (300 MHz, DMSO/CCl_4_, 1/3) *δ* 1.40 (t, *J* = 7.1 Hz, 3H, CH_2_CH_3_), 2.15−2.27 (m, 2H, 7-CH_2_), 3.15 (t, *J* = 7.5 Hz, 2Н, 6-CH_2_), 3.45 (t, *J* = 7.5 Hz, 2H, 8-CH_2_), 4.31 (q, *J* = 7.1 Hz, 2H, CH_2_CH_3_), 6.52 (br s, 2H, NH_2_), 7.35−7.48 (m, 3H, Ph), 7.77−7.81 (m, 2H, Ph). Anal. Calcd. for C_19_H_18_N_2_O_2_S: C 67.43; H 5.36; N 8.28 %. Found: C 67.77; H 5.54; N 8.51 %.

*General procedure for the synthesis of compounds 6a−e*

A mixture of compound **5a**−**d**,**f** (2 mmol) and hydrazine hydrate (1 g, 20 mmol) [or pyrrolidine (0.18 mL, 2.2 mmol) for compound **6e**] in absolute ethanol (25 mL) was refluxed for 10 h. The reaction mixture was cooled, water (50 mL) was added. The separated crystals were filtered off, washed with water, dried, and recrystallized from ethanol.

*1-Amino-5-phenyl-7,8-dihydro-6H-cyclopenta[d]furo[2,3-b]pyridine-2-carbohydrazide (6a).* Light yellow solid, yield 74 %, mp 228−230 ^o^C; IR *ν*/cm^−1^: 1634 (C=O), 3253, 3349, 3437 (NH, NH_2_). ^1^H NMR (300 MHz, DMSO/CCl_4_, 1/3) *δ* 2.22 (m, 2H, 7-CH_2_), 3.14 (t, *J* = 7.3 Hz, 2H, 6-CH_2_), 3.35 (t, *J* = 7.5 Hz, 2H, 8-CH_2_), 4.20 (br s, 2H, NHNH_2_), 5.57 (br s, 2H, NH_2_), 7.33‒7.54 (m, 3H, Ph), 7.70‒7.79 (m, 2H, Ph), 9.05 (br, 1H, NH). ^13^C NMR (75 MHz, DMSO/CCl_4_, 1/3) δ 25.62, 30.51, 31.56, 110.66, 125.20, 127.52, 127.63, 128.04, 131.89, 134.30, 139.34, 149.80, 150.37, 158.13, 160.73. Anal. Calcd. for C_17_H_16_N_4_O_2_: C 66.22; H 5.23; N 18.17 %. Found: C 66.59; H 5.45; N 18.43 %.

*1-Amino-5-(2-furyl)-7,8-dihydro-6H-cyclopenta[d]furo[2,3-b]pyridine-2-carbohydrazide* **(6b).** Light yellow solid, yield 83 %, mp 273−275 ^o^C; IR *ν*/cm^−1^: 1620 (C=O); 3318, 3345 3437 (NH, NH_2_). ^1^H NMR (300 MHz, DMSO/CCl_4_, 1/3) *δ* 2.21 (m, 2H, 7-CH_2_), 3.19 (t, *J* = 7.4 Hz, 2H, 6-CH_2_), 3.30 (t, *J* = 7.5 Hz, 2H, 8-CH_2_), 4.37 (br, 2H, NHNH_2_), 5.74 (br s, 2H, NH_2_), 6.68 (dd, *J* = 3.6, 1.7 Hz, 1Н, 4-CH_furyl_), 7.06 (d, *J* = 3.6 Hz, 1Н, 3-CH_furyl_), 7.88 (d, *J* = 1.7 Hz, 1Н, 5-CH_furyl_), 9.32 (br, 1H, NH). ^13^C NMR (75 MHz, DMSO/CCl_4_, 1/3) δ 24.66, 30.38, 30.94, 110.57, 110.90, 112.15, 125.27, 130.38, 134.42, 141.17, 144.37, 150.76, 156.12, 157.72, 160.81. Anal. Calcd. for C_15_H_14_N_4_O_3_: C 60.40; H 4.73; N 18.78 %. Found: C 60.71; H 4.93; N 19.02 %.

*1-Amino-5-methyl-6,7,8,9-tetrahydrofuro[2,3-c]isoquinoline-2-carbohydrazide (6c).*

Light yellow solid, yield 75 %, mp 270−272 ^o^C; IR *ν*/cm^−1^: 1613 (C=O), 3283 (NH, NH_2_). ^1^H NMR (300 MHz, DMSO/CCl_4_, 1/3) *δ* 1.77–1.91 (m, 4H, 7,8-CH_2_), 2.42 (s, 3H, CH_3_), 2.62–2.67 (m, 2H, 6-CH_2_), 3.15–3.20 (m, 2H, 9-CH_2_), 4.20 (br, 2H, NHNH_2_), 5.52 (br s, 2H, NH_2_), 8.97 (br, 1H, NH). Anal. Calcd. for C_13_H_16_N_4_O_2_: C 59.99; H 6.20; N 21.52 %. Found: C 60.34; H 6.38; N 21.75 %.

*1-Amino-5-phenyl-7,8-dihydro-6H-cyclopenta[d]thieno[2,3-b]pyridine-2-carbohydrazide* **(6d).** Light yellow solid, yield 77 %, mp 220−222 ^o^C; IR *ν*/cm^−1^: 1630 (C=O), 3247, 3364, 3451 (NH, NH_2_). ^1^H NMR (300 MHz, DMSO/CCl_4_, 1/3) *δ* 2.18 (m, 2H, 7-CH_2_), 3.12 (t, *J* = 7.5 Hz, 2H, 6-CH_2_), 3.44 (t, *J* = 7.6 Hz, 2H, 8-CH_2_), 4.40 (br, 3H, NHNH_2_), 6.49 (br s, 2H, NH_2_), 7.31‒7.48 (m, 3H, Ph), 7.70‒7.82 (m, 2H, Ph). ^13^C NMR (75 MHz, DMSO/CCl_4_, 1/3) δ 24.88, 31.14, 31.61, 97.67, 122.36, 127.46, 127.79, 128.16, 132.91, 139.21, 145.73, 149.56, 153.35, 157.22, 165.35. Anal. Calcd. for C_17_H_16_N_4_OS: C 62.94; H 4.97; N 17.27 %. Found: C 63.27; H 5.18; N 17.52 %.

*5-(2-Furyl)-2-(pyrrolidin-1-ylcarbonyl)-6,7,8,9-tetrahydrofuro[2,3-c]isoquinolin-1-amine* *(6e).* Light yellow solid, yield 85 %, mp 228−230 ^o^C; IR *ν*/cm^−1^: 1643 (C=O), 3434, 3342 (NН_2_). ^1^H NMR (300 MHz, DMSO/CCl_4_, 1/3) *δ* 1.75–2.15 (m, 8H, 7,8-CH_2_, 2CH_2_-C_4_H_8_N), 3.00–3.10 (m, 2H, 6-CH_2_), 3.24–3.33 (m, 2H, 9-CH_2_), 3.38−4.00 (m, 4H, N(CH_2_)_2_), 5.78 (br s, 2H, NH_2_), 6.54 (dd, *J* = 3.5, 1.8 Hz, 1H, 4-CH_furyl_), 6.96 (dd, *J* = 3.5, 0.8 Hz, 1H, 3-CH_furyl_), 7.62 (dd, *J* = 1.8, 0.8 Hz, 1H, 5-CH_furyl_). Anal. Calcd. for C_20_H_21_N_3_O_3_: C 68.36; H 6.02; N 11.96 %. Found: C 68.72; H 6.19; N 12.18 %.

*Confocal microscopy*

Vero cells seeded on glass coverslips (1 × 10^5^/cm^2^), either mock-infected or infected with the ASFV Ba71V (1 TCID50/cell), were exposed to the compound (100 µM) or to solvent alone (DMSO); exposure to drug or solvent was for either 8 or 16 hours before collection. At these time points, cells were fixed in 3.7% paraformaldehyde in HPEM buffer [30 mM HEPES (4-(2-hydroxyethyl)-1-piperazineethanesulfonic acid) containing 65 mM PIPES (piperazine-N,N′-bis 2-ethanesulfonic acid), 10 mM EGTA (ethylene glycol tetraacetic acid) and 2 mM MgCl_2_; pH 6.9] plus 0.5% (v/v) Triton X-100 for 10 min, at room temperature, before incubation with antibodies. All washes were done with PBS containing 0.05% Triton X-100, and antibodies were diluted in PBS containing fetal bovine serum (5% v/v; blocking agent), 0.05% (v/v) Triton X-100 and 0.1% (w/v) sodium azide.

The immunostaining of ASFV-pI215L and ASFV-infected cells were achieved by incubation with two in-house primary antibodies: mouse anti-ASFV-PI215L and anti-ASFV swine serum directly conjugated to fluorescein isothiocyanate (FITC; 1:100, 1 h, RT) as previously described [47, 48]. The affinity purified Alexa Fluor 568-conjugated goat anti-mouse secondary antibody (Thermo Fisher Scientific; A-11031; 1:150) was used to detect the ASFV-pI215L, whereas the monoclonal rat anti-tubulin antibody (Abcam; ab 6160; 1:1000) and the Alexa Fluor 488-conjugated donkey anti-rat (Thermo Fisher Scientific; 1:150; A-21208) were used to evaluate the effects of the compound 6b on the tubulin network. For double-immunostaining experiments primary antibodies were mixed to the specified final concentrations. Between each antibody incubation, cells were washed twice with PBS for 5 minutes and once with PBST (0.1% v/v for 5 minutes). All incubations were performed in a dark humidified chamber to prevent fluorochrome fading. After immunolabeling, cell nucleus and viral factories were stained with DAPI (4′,6-diamidino-2-phenylindole, 0.5 μg/mL) and coverslips were mounted in Vectashield (Vector Laboratories Inc., Burlingame, CA, USA) before analysis by fluorescence microscopy.

In some experiments, ASFV-infected cells were exposed to EdU (5-ethynyl-2′-deoxyuridine; 25 microM) for 15 min before collection; cells exposed to solvent (DMSO) served as controls. EdU incorporated into replicating DNA was detected with Click-iT EdU 6-FAM azide kit following the manufacturer's instructions (Baseclick GmbH, Neuried, Germany).

Samples were examined using a Zeiss 710 confocal microscope (Carl Zeiss, Jena, Germany) equipped with lasers giving excitation lines at 405, 488 and 561 nm. Data from the channels were collected separately using narrow-band-pass filter settings. In multiple staining experiments, the laser intensities were adjusted to avoid bleed-through between channels. Data was collected with two- to four-fold averaging at resolution of 1024 × 1024 pixels using pinhole settings between 1.05 and 1.10 airy units. Data sets were processed using Zeiss 710 version 2.8 software package and were subsequently exported for preparation for printing using Adobe Photoshop, version CS5.1 (Adobe Systems, Inc., San Jose, USA).

*Histology and blood cells analysis*

All animals (white mice; both sexes; 20-25g; n_control_ = 5; n_exp_ = 4 for each dose) were sacrificed at 25 days post-administration according to the institutional and national guidelines for laboratory animals. Tissue samples from liver and spleen were fixed in 10 % buffered formalin (pH 7.2) for 24 hours. After fixation, samples were dehydrated through a graded series of alcohols, washed with xylol and embedded in paraffin wax by a routine technique for light microscopy. For anatomopathological examination, wax-embedded samples were cut (Microm HM 355, 5 µm) and stained with hematoxylin and eosin according to the manufacturer’s protocol (Sigma-Aldrich, Germany). The histological examination was carried out using a light microscope.

All blood samples were collected from the jugular vein of mice. Fresh blood was used in preparing the blood smears by routine methods. For nucleated blood cells analysis, slides were fixed in pure methanol and stained by Giemsa modified solution (azure B/azure II, eosin and methylene blue) according to the manufacturer’s protocol (Sigma-Aldrich, Germany), Slides were fixed for 10 minutes in methanol, immersed for 45 minutes in Giemsa solution and rinsed with distilled water. White blood cells were examined under the light microscope at ×1250 in a random sequence. At least 200 white blood cells in each sample were evaluated for cell types.

**Results**

**
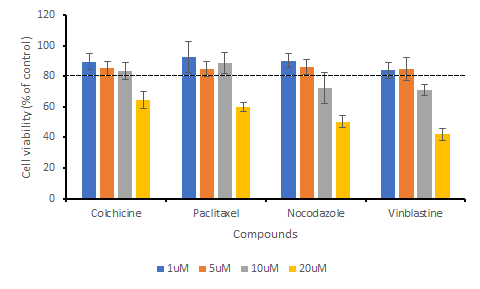
**

**Figure 1S. Cytotoxicity of microtubule-targeting agents on Vero cells**. The cytotoxicity of drugs was evaluated by MTT assay at 24 h of incubation. Drug concentrations were considered non-cytotoxic if cell viability was higher than 80% of control (dash line).

**
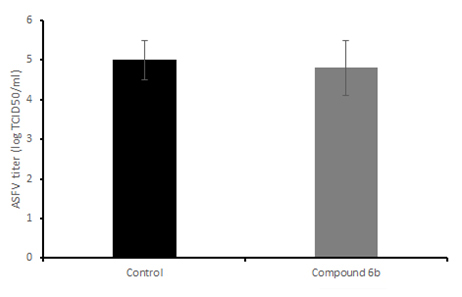
**

**Figure S2. Direct effect of compound 6b on extracellular ASFV particles.** Virus was co-incubated with compound **6b** at 100 µM concentration at room temperature. ASFV titers were determined by CPE-based assay.


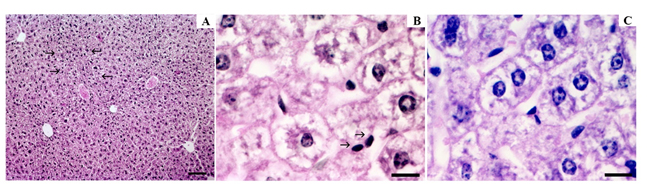


**Figure S3. Liver sections stained by H&E.** (A) Development of hydropic degeneration in hepatocytes. Hydropic degeneration in peripheral part of classic lobuli (indicated by arrows). Scale is 50µm. (B) Central localization of hepatocyte nucleus in vacuolated cells. Scale is 10µm. (C) Binuclear hepatocytes. Scale 10µm.

**Table S1. The half-life of compound 6b and tubulin-targeting drugs predicted by ICM software.**

| **Compound** | **PubChem ID** | **Plasma Half-life (hours)** |
| --- | --- | --- |
| Compound 6b | - | 1.425146 |
| Colchicine | 6167 | 6.024944 |
| Paclitaxel | 36314 | 3.32472 |
| Nocodazole | 4122 | 2.471672 |
| Vinblastine | 13342 | 1.914483 |

**Table S2. Composition of blood cells.**

| **Cell types** | **The percent (%) of cells** | |
| --- | --- | --- |
|  | **Control (mean** ± SD) | **Compound 6b (mean** ± SD) |
| Erythroblasts | 29.5 ± 6.1 | 26.2 ± 5.1 |
| Lymphoblasts | 6.3 ± 1.1 | 4.9 ± 1.0 |
| Lymphocytes | 28.4 ± 5.4 | 23.2 ± 4.9 |
| Monoblasts | 2.1 ± 1.0 | 3.5 ± 1.7 |
| Monocytes | 2.7 ± 1.0 | 2.6 ± 1.1 |
| Myeloids | 4.8 ± 0.9 | 5.3 ± 1.6 |
| Metamyelocytes | 3.6 ± 1.8 | 6.3 ± 1.1* |
| Band neutrophils | 9.1 ± 2.2 | 12.8 ± 2.7* |
| Segmented neutrophils | 9.5 ± 2.7 | 11.4 ± 2.6* |
| Eosinophils | 3.3 ± 1.4 | 3.2 ± 1.2 |
| Basophils | 0.2 ± 0.1 | 0.1 ± 0.1 |
| Megakaryocytes | 0.3 ± 0.2 | 0.3 ± 0.1 |
| Macrophages | 0.2 ± 0.1 | 0.2 ± 0.3 |
| Pathological forms | - | 0.1 ± 0.2 |

*significant increase compared with control (p<0.05 – p<0.01)

**S1 video. MD simulation of compound 6b in the taxane site.** The M-loop is rearranged into a short alpha-helix and changes of secondary structures are color-coded: loop - cyan, 3-10 helix - blue, alpha-helix – magenta.
